# Prägnanz in visual perception

**DOI:** 10.3758/s13423-023-02344-9

**Published:** 2023-10-03

**Authors:** Eline Van Geert, Johan Wagemans

**Affiliations:** Laboratory of Experimental Psychology, Department of Brain and Cognition, Faculty of Psychology and Educational Sciences, KU Leuven, Tiensestraat 102 - box 3711, 3000, Leuven, Belgium

**Keywords:** Good Gestalt, Gestalt psychology, Perceptual organization, Figural goodness, Simplicity

## Abstract

How do we perceptually and cognitively organize incoming stimulation? A century ago, Gestalt psychologists posited the law of Prägnanz: psychological organization will always be as ‘good’ as possible given the prevailing conditions. To make the Prägnanz law a useful statement, it needs to be specified further (a) what a *‘good’* psychological organization entails, (b) *how* the Prägnanz tendency can be realized, and (c) *which conditions* need to be taken into account. Although the Gestalt school did provide answers to these questions, modern-day mentions of *Prägnanz* or good Gestalt often lack these clarifications. The concept of *Prägnanz* has been (mis)understood in many different ways, and by looking back on the rich history of the concept, we will attempt to present a more fine-grained view and promote a renewed understanding of the central role of Prägnanz in visual perception and beyond. We review Gestalt psychology’s answers to the questions listed above, and also discuss the four main uses of the Prägnanz concept in more detail: (a) a Prägnanz tendency in each organizational process, (b) Prägnanz as a property of a Gestalt, (c) Prägnanz steps as internal reference points, and (d) Prägnanz in relation to aesthetic appreciation. As a key takeaway, Prägnanz is a multifaceted Gestalt psychological concept indicating the “goodness” of an experienced organization. Both the removal of unnecessary details and the emphasis on characteristic features of the overall organization compared to a reference organization can contribute to the emergence of a ‘better’ Gestalt. The stimulus constellation is not the only factor in determining the goodness of an organization, also the stimulus’ interaction with an individual in a specific spatial and temporal context plays a role. Taking the ideas on Prägnanz as a generative framework and keeping the original Gestalt psychological context in mind, future research on perceptual organization can improve our understanding of the principles underlying psychological organization by further specifying how different organizational principles interact in concrete situations. **Public significance statement:** This paper reviews what a ‘good’ psychological organization entails, and how the incoming stimulation is clarified in human perception to achieve the best possible psychological organization. The review debunks common misconceptions on the meaning of “goodness” and synthesizes the most important perspectives and developments on “goodness” from its conception until now.

## Prägnanz in visual perception

**Fig. 1:**
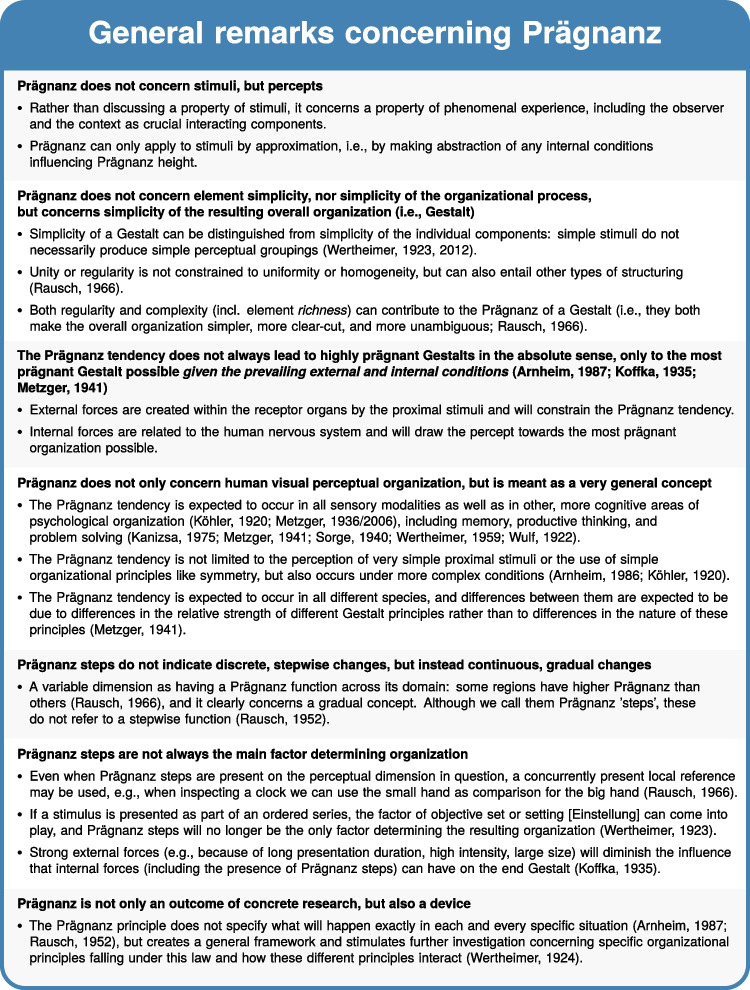
Important general remarks concerning the Prägnanz concept. Figure licensed under CC BY 4.0 by the authors. Retrieved from https://doi.org/10.6084/m9.figshare.21977504

How do we perceptually and cognitively organize incoming stimuli? A century ago, Gestalt psychologists posited the law of Prägnanz: psychological organization will always be as ‘good’ as possible given the prevailing conditions. Although very commonly referred to in journal article introduction and discussion sections on perceptual organization, further clarification of (a) what a *‘good’* psychological organization entails, (b) *how* the Prägnanz tendency can be realized, and (c) *which conditions* need to be taken into account, is often lacking in these modern-day references to Prägnanz. In addition, the Prägnanz concept has been (mis)understood in many different ways (cf. Fig. 1). For example, in more recent years, *Prägnanz* has often been equated with element simplicity and the minimum principle: It was assumed that a visual *stimulus* is prägnant when it consists of *few elements*. The roots of the concept suggest a different, much richer and more complex interpretation, however. Rather than discussing a property of *stimuli*, it concerns a property of phenomenal experience, including the observer and the context as crucial interacting components. Moreover, not simplicity of the elements is central to the concept of *Prägnanz*, but simplicity of the whole, complete configuration as perceived by the observer, i.e., *simplicity of the Gestalt*. Part of the narrowing and misconceptions concerning Prägnanz may be due to many of the original sources – written in German – not being translated in English. Furthermore, if they were translated, this often happened only partially, or rather recently (e.g., Metzger , 1936/2006; Wertheimer et al. , 2012; Wagemans , 2015; cf. also Wagemans, Elder, et al., 2012).

**Fig. 2:**
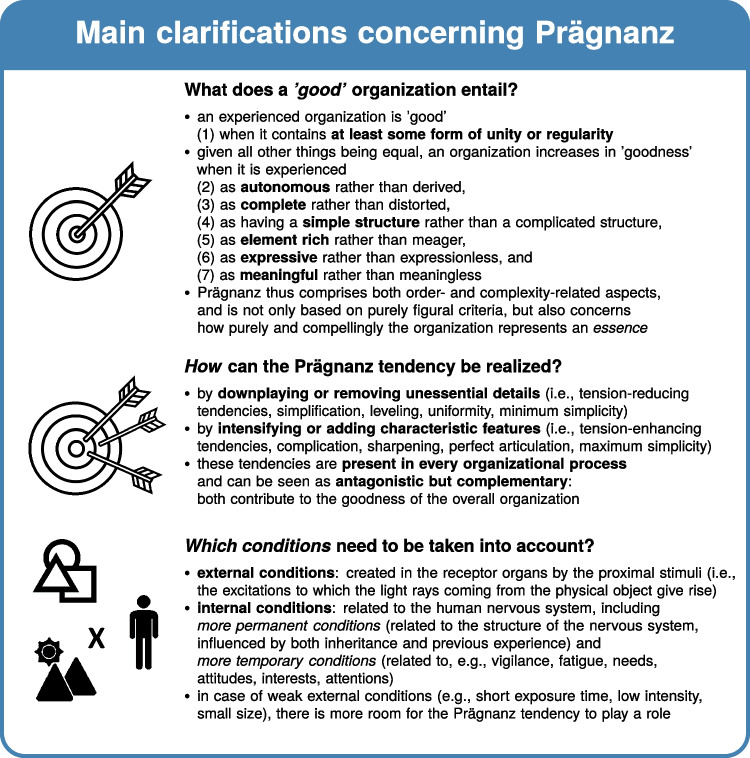
Main clarifications concerning Prägnanz. *Note.* The icons in this Figure were adapted from the following CC BY licensed icons from the Noun Project: target by Support Designs, head by Maxim Kulikov, shapes by Andrejs Kirma, and landscape by Baboon designs. Figure licensed under CC BY 4.0 by the authors. Retrieved from https://doi.org/10.6084/m9.figshare.21977522

**Fig. 3:**
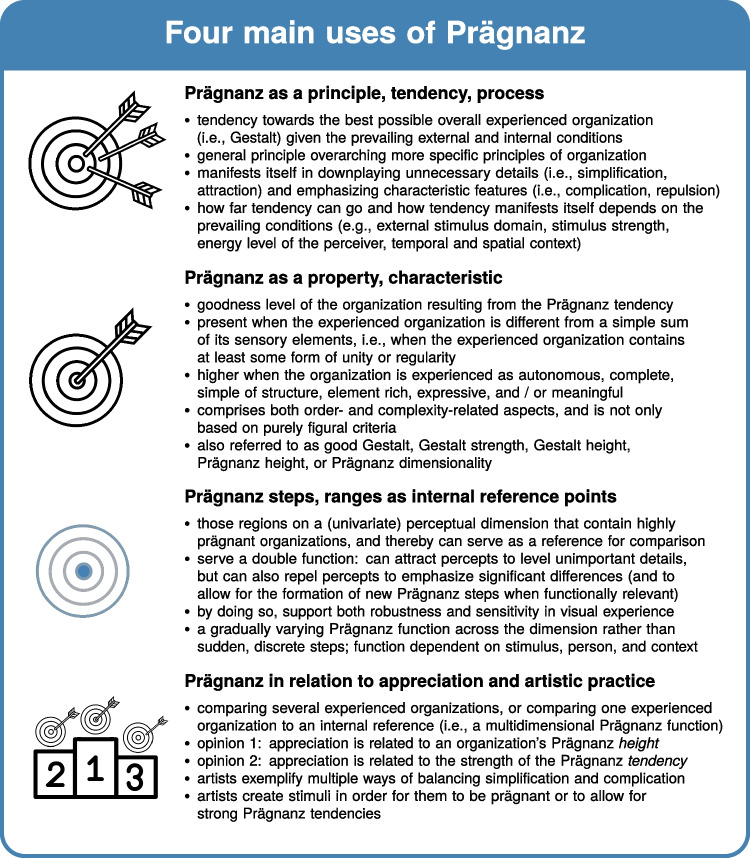
Four main uses of Prägnanz. *Note.* The icons in this Figure were adapted from the following CC BY licensed icons from the Noun Project: target by Support Designs and podium by Prettycons. Figure licensed under CC BY 4.0 by the authors. Retrieved from https://doi.org/10.6084/m9.figshare.21977540

By looking back on the history of Prägnanz, we attempt to present a more fine-grained view and promote a renewed understanding of the central role of Prägnanz in visual perception and beyond. We review Gestalt psychology’s answers to the questions listed above (cf. Fig. 2), and discuss the four main uses of the Prägnanz concept in more detail: (a) a Prägnanz tendency in each organizational process, (b) Prägnanz as a property of a Gestalt, (c) Prägnanz steps as internal reference points, and (d) Prägnanz in relation to aesthetic appreciation (cf. Fig. 3). Furthermore, we counter common critiques and reject alternative conceptualizations of Prägnanz by pointing back to the original intentions of Gestalt psychologists when positing the Prägnanz principle. Importantly, the Prägnanz principle was not meant as a magical one-fits-all solution, and it should be seen not only as an outcome of concrete research results but also as a device to stimulate further research (Wertheimer, 1924/1999): by using Gestalt theory and the Prägnanz principle as a generative framework for future research, and by studying more specific principles of organization and their interaction in concrete cases (Rausch, 1966), we can come to a better understanding of the principles underlying psychological organization.

## Prägnanz in all its facets

At the end of the nineteenth and the beginning of the twentieth century, the widespread view on perception (and science in general) was elementaristic: researchers believed that a perceptual experience was reducible to its elementary sensations. This is, in fact, still the dominant view in most areas of experimental psychology and cognitive (neuro)science (which also demonstrates why a careful review and analysis of the alternative view is still relevant today). Wertheimer (1922, 1924/1999) and the Berlin Gestalt school questioned the immediate givenness of independent elementary sensations and instead posited the primacy of the whole and direct influences of the whole on the perception of the elements: our experience of every individual element is, from the start, influenced by our organization of the whole. For example, in a melody, a particular tone will be experienced differently depending on which role this tone has in the melody (e.g., leading tone vs. tonic; Wertheimer , 1922, 1924/1999). Uncovering the principles guiding the spontaneous self-organization of the phenomenal field – without dissolving the perceptual experience by taking an item-per-item approach – is a key task for Gestalt psychology, and will lead to a better understanding of groupings and divisions in perceptual experience (Ellis, 1938; Wertheimer, 1922, 1923; Wertheimer et al., 2012).

Gestalt psychology thus takes it as its core task to reveal the principles that govern spontaneous self-organization in our phenomenal experience, including visual perception. Koffka (1935) designates the *Prägnanz principle* (i.e., the tendency towards the best possible overall organization given the prevailing conditions) as the most important principle to guide research on perceptual organization. This tendency towards Prägnanz of Gestalts was seen as the general principle overarching more specific principles of organization (e.g., grouping by proximity, similarity, good continuation). Under weak stimulus conditions, this tendency gets more room to play a role and can even lead to tangible dislocations and distortions compared to the external stimulation. Both the removal or softening of unnecessary details (i.e., simplification, leveling) and the addition or emphasis on characteristic features of the Gestalt organization (i.e., complication, sharpening) can take place and lead to a ‘better’ overall organization.

In addition to the Prägnanz tendency present in every organizational process, the term *Prägnanz* is also used *as a property* to characterize the organization or Gestalt *resulting* from this organizational process. To be ‘prägnant’, a psychological organization needs to be different from a simple sum of its elements: it has to be a Gestalt. In contrast to von Ehrenfels and the Austrian Gestalt psychological school, who defined Gestalt as a quality or characteric of an ensemble of items (Smith, 1988), Wertheimer (1922) and the other members of the Berlin Gestalt school defined a Gestalt as an ensemble of items that mutually support and determine one another (Sundqvist, 2003). Following the Berlin school, we could thus say that something is a Gestalt when our phenomenal experience is different from the experience of a pure sum of sensory elements — different from a pure and-summation (Koffka, 1935; Smith, 1988). This criterion for a phenomenal experience to contain at least some form of unity or regularity also comes back as the first and only necessary criterion in Rausch ’s (1966) specification of Prägnanz (cf. the section on “Rausch ’s (1966) Prägnanz aspects” below). In addition, Prägnanz can increase when the organization is perceived as autonomous rather than derived, complete rather than disrupted, simple of structure rather than complicated of structure, element rich rather than element poor, expressive rather than expressionless, and meaningful rather than meaningless. A psychological organization can thus be ‘good’ for several reasons, not only based on purely figural criteria. Moreover, Prägnanz is increased not only by increasing unity or regularity of the whole, overall organization, but also by increasing intricacy of its underlying components, of its relation between structure and meaning, and of its interaction with already existing knowledge structures in the organism.

Psychological organizations that excel in their *Prägnanz* can influence our phenomenal experience of new incoming information: they serve as a reference to which the input is internally compared. The term *Prägnanz steps* [*Prägnanzstufen*] is used in this context to refer to prägnant forms that serve *as reference regions* on a univariate dimension (e.g., the right angle in the realm of all possible angles). These Prägnanz steps serve a double function: on the one hand, assimilation to these Prägnanz steps may occur (especially when the external stimulus factors are weak); on the other hand, these reference points can increase sensitivity in their vicinity (to increase the ability to notice small deviations from the Prägnanz step). In that sense, Prägnanz steps support both robustness and sensitivity in visual experience: under weak stimulus conditions (i.e., under uncertainty) or when a specific difference is deemed unimportant, their stimulating effect to adhere to the best possible organization will dominate; under clear stimulus conditions and when a specific difference is significant, their influence on discrimination sensitivity in those regions where deviations matter most (i.e., close to the Prägnanz steps) will dominate. Their stimulating effect on discrimination sensitivity also allows for the formation of new reference levels in between existing ones when this becomes behaviorally or functionally useful. Rather than a binary distinction between reference points and non-reference points, a gradual Prägnanz function applies to each variable dimension, with some regions showing higher Prägnanz than others (e.g., the right angle as a higher Prägnanz step than the Prägnanz steps of sharp and obtuse angles; Rausch , 1966). The course of this Prägnanz function per domain and dimension can differ between individuals and contexts. For example, experience may elicit the formation of more and narrower Prägnanz steps on a dimension (Rausch, 1966; Wertheimer, 1923): individuals who deal with angles frequently (e.g., designers, architects) may have additional Prägnanz steps around 45^∘^ angles (besides 0^∘^ and 90^∘^ angles), and their Prägnanz steps may be narrower (e.g., only ranging from 89^∘^ to 91^∘^ around 90^∘^ angles instead of ranging from 87^∘^ to 93^∘^).

When we psychologically organize incoming stimulation, we can *not only* describe or classify the experienced organization in a purely structural or semantic sense, but we can also evaluate our aesthetic experience of this organization. Since perceptual processing of the incoming information is necessary to be able to aesthetically evaluate our percept, the close relation between perception and *aesthetics* cannot be neglected. von Ehrenfels (1916, 1922) called beauty nothing else than Gestalt height, which he defined as the product of unity (of the whole) and multiplicity (of the parts). Unity-in-variety is also a major principle in design (e.g., Post et al., 2016). Moreover, Koffka (1940) called perception artistic and both Metzger (1941) and Arnheim (1975) noticed the presence of simplification and complication tendencies in artistic practice. In our view, aesthetic appreciation may arise together with a conscious increase in Prägnanz (i.e., the strength of the experienced Prägnanz tendency). This view also relates closely to other accounts of aesthetic appreciation, including the predictive processing accounts of Van de Cruys and Wagemans (2011) and Chetverikov and Kristjánsson (2016) as well as the focus on pleasure by insights into Gestalt proposed by Muth and Carbon (2013, 2016; Muth et al., 2013). On the other hand, aesthetic appreciation could also be based on the absolute level of Prägnanz experienced, and this does not necessarily relate to the strength of the Prägnanz *tendency*. Nevertheless, both views may act in complementary ways as well.

In what follows, we will discuss the four main uses of the Prägnanz concept mentioned above (cf. Fig. 3) in more detail: (a) a Prägnanz tendency in each organizational process, (b) Prägnanz as a property of a Gestalt, (c) Prägnanz steps as internal reference points, and (d) Prägnanz in relation to aesthetic appreciation. Although these uses overlap in many ways, we distinguish them here as they all focus on a different facet of the overall concept. A full understanding of Prägnanz encompasses all of these facets and their interrelations in a compelling whole, however.

**Fig. 4:**
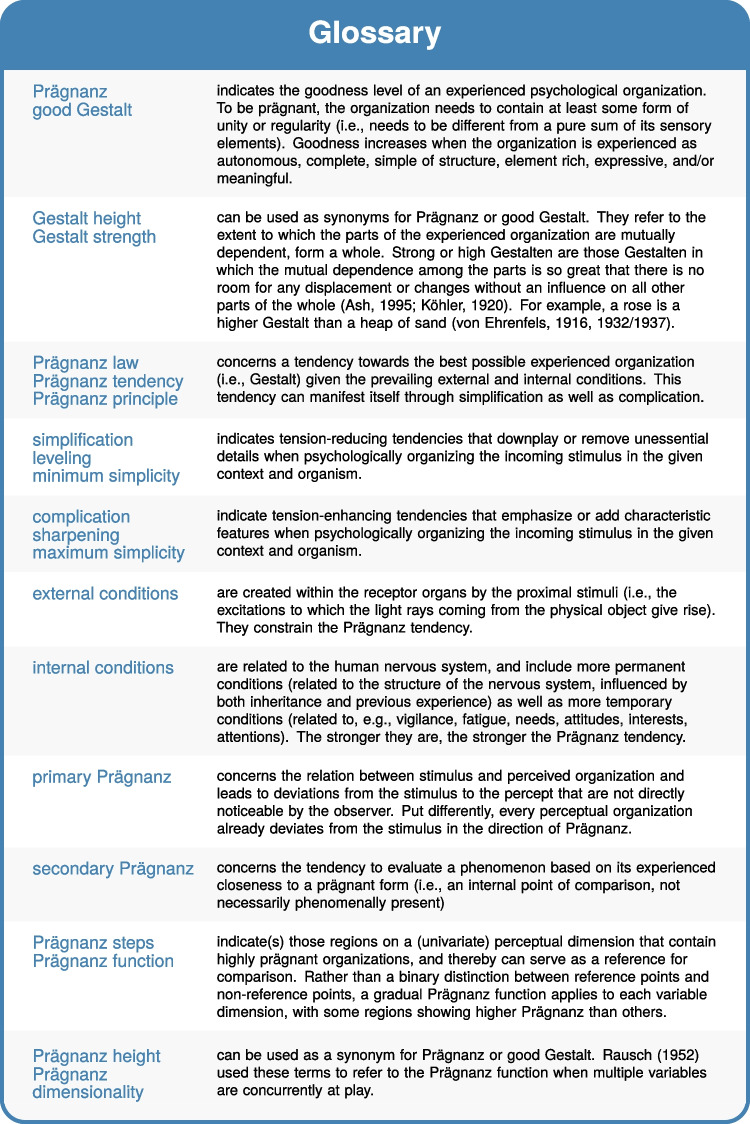
Glossary of Prägnanz-related terminology. Figure licensed under CC BY 4.0 by the authors. Retrieved from https://doi.org/10.6084/m9.figshare.21977546

**Fig. 5:**
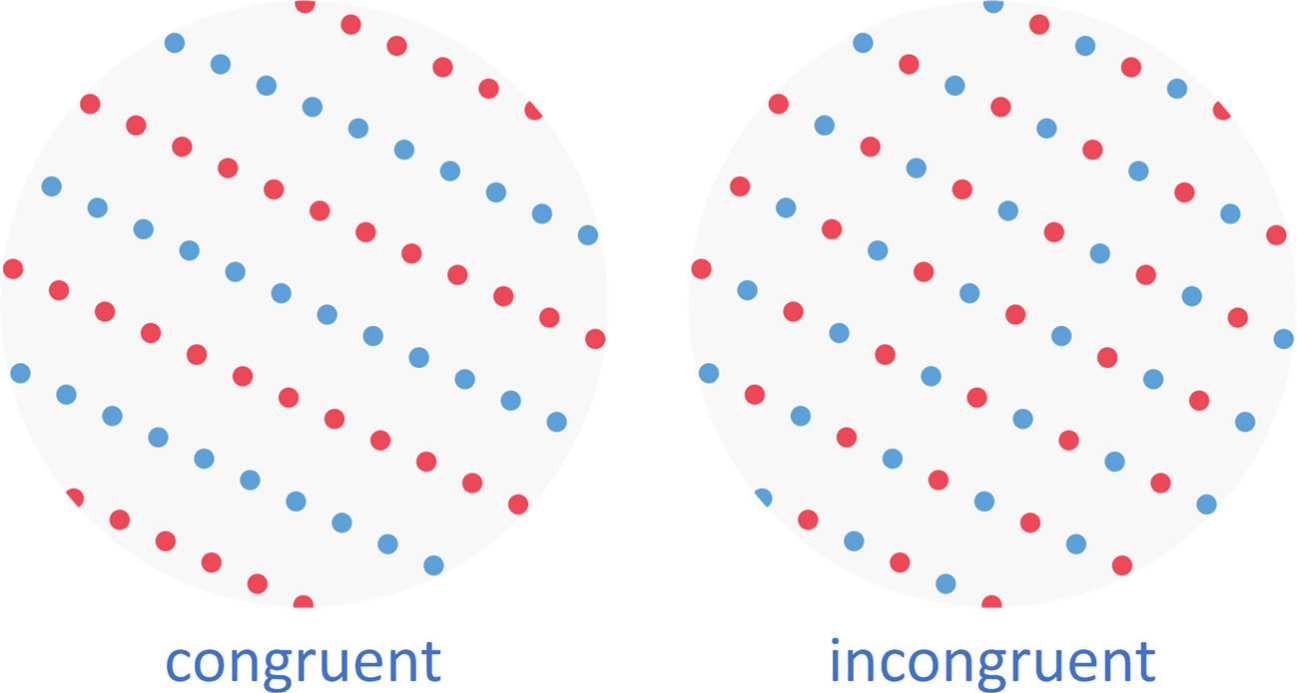
Example of a dot lattice in which proximity grouping and color similarity grouping are either congruent or incongruent. These lattices were created using the OCTA toolbox (Van Geert, Bossens, & Wagemans, 2023). Figure licensed under CC BY 4.0 by the authors. Retrieved from https://doi.org/10.6084/m9.figshare.21977558

### A Prägnanz tendency present in each organizing process

When we view Prägnanz as a tendency present in each organizing process, we speak of the **Prägnanz law**, the **Prägnanz principle**, or the **Prägnanz tendency** (cf. Fig. 4). Etymologically, Prägnanz derives from the German verb ‘prägen’ (i.e., to mint a coin) and the Latin verb ‘premere’ (i.e., to press a point), and therefore Prägnanz refers to being sharply grasping, unambiguous, clear, or distinct (Arnheim, 1975; Wagemans, 2018)^1^. The Prägnanz tendency thus concerns a tendency, present in all forms of psychological organization, to evolve in the direction of a more clear-cut overall organization (i.e., a better Gestalt). This is equivalent to evolving in the direction of minimal structural energy to arrive at a stable organization. The first written mention of Prägnanz as a tendency comes from Wertheimer (Schumann, 1914), who described his success in ascertaining, under several Gestalt laws of a general nature, *“ein Gesetz der Tendenz zum Zustandekommen einfacher Gestaltung (Gesetz ‘zur Prägnanz der Gestalt’)”* (i.e., a law of the tendency to come towards a simple Gestalt or a law towards Prägnanz of a Gestalt; Schumann, 1914, p. 149). Importantly, simplicity of the Gestalt can be distinguished from simplicity of the individual components: simple stimuli do not necessarily produce simple perceptual groupings (Wertheimer, 1923; Wertheimer et al., 2012).

Wertheimer (1923) proposed the law of good Gestalt as an overarching law of which the other Gestalt laws are special cases: When forming a percept and a grouping, the “best” overall organization, the simplest “whole” will win, and each specific law (e.g., proximity, similarity, good continuation; cf. also Wagemans , 2018; Wagemans, Elder, et al., 2012; Wertheimer , 1923) gives an indication of what the “best” grouping will be. For example, the law of similarity indicates that there is a tendency for uniformly colored parts to group together. Following the law of similarity, the “best”, most simple overall organization will thus be the one with uniformly colored components (e.g., red parts grouping together, blue parts grouping together; Wertheimer , 1923), but this may oppose the best organization according to another Gestalt principle (e.g., the law of proximity).

What happens if several Gestalt principles are concurrently at play (e.g., Fig. 5)? If several Gestalt principles are working in the same direction, this will lead to stronger inner cohesion and sharper segmentation (Metzger, 1941), in other words, to a better, more prägnant Gestalt. Conflicting Gestalt factors will yield one of five possible states: (1) one of the principles is stronger and wins; (2) the result is ambiguous and there is switching between two possible organizations; (3) the result is unclear, chaotic; (4) a richer organization forms in which both Gestalt factors play a role; or (5) one of the Gestalt factors wins but the end Gestalt is slightly changed based on the other Gestalt factor (Metzger, 1941). Inter- and intra-individual differences will play an important role in determining whether (3) or (4) will be the case (e.g., comprehension capacity *[Fassungsvermögen]*, expertise, energy level, mood; Metzger, 1941). Importantly, the law of Prägnanz does not specify what will happen exactly in each and every specific situation (Arnheim, 1987; Rausch, 1952), but it creates a general framework and stimulates further investigation concerning specific organizational principles falling under this law and how these different principles interact (Wertheimer, 1924/1999).

Köhler (1920) focused on the equivalence between Wertheimer’s tendency towards Prägnanz of a Gestalt and the physical tendency towards minimal structural energy (attained when in a stable, stationary state). In processes ending in a stable state, there is a tendency in the direction of minimal structural energy, i.e., a tendency to achieve minimal structural energy in the end state or resulting organization given what is possible under the prevailing conditions (Köhler, 1920). As only the final structure or organization – and not the corresponding energy level – is available in phenomenal experience, we cannot determine whether a perceived organization corresponds to the minimal energy level based on phenomenology alone. One could only infer whether the Prägnanz principle of minimal structural energy is realized in the nervous system for a specific perceived organization if a simple relationship existed between the energy level and the perceived organization (Köhler , 1920; cf. also Pepperell , 2018).

The best-known classical description of the law of *Prägnanz* is probably the one by Koffka (1935) in his English book on the “Principles of Gestalt Psychology”: “psychological organization will always be as ‘good’ as the prevailing conditions allow” (p. 110). In this definition, the term ‘good’ is undefined, but entails properties such as regularity, symmetry, simplicity, and others (Koffka , 1935, p. 110). Koffka (1935) presents the law in the context of his discussion on finding the true solution to the question “Why do things look as they do?”. False solutions mentioned include “because things are what they are” (veridicality; cf. Box 1) and “because the proximal stimuli [i.e., the excitations to which the light rays coming from the physical object give rise] are what they are”. Things look as they do “because of the field organization to which the proximal stimulus distribution gives rise. [...] It means that we have to study the laws of organization” (Koffka , 1935, p. 98). The law of *Prägnanz* is mentioned as the main principle to guide research on psychophysical^2^ (i.e., perceptual) organization (Koffka , 1935, p. 110).

 
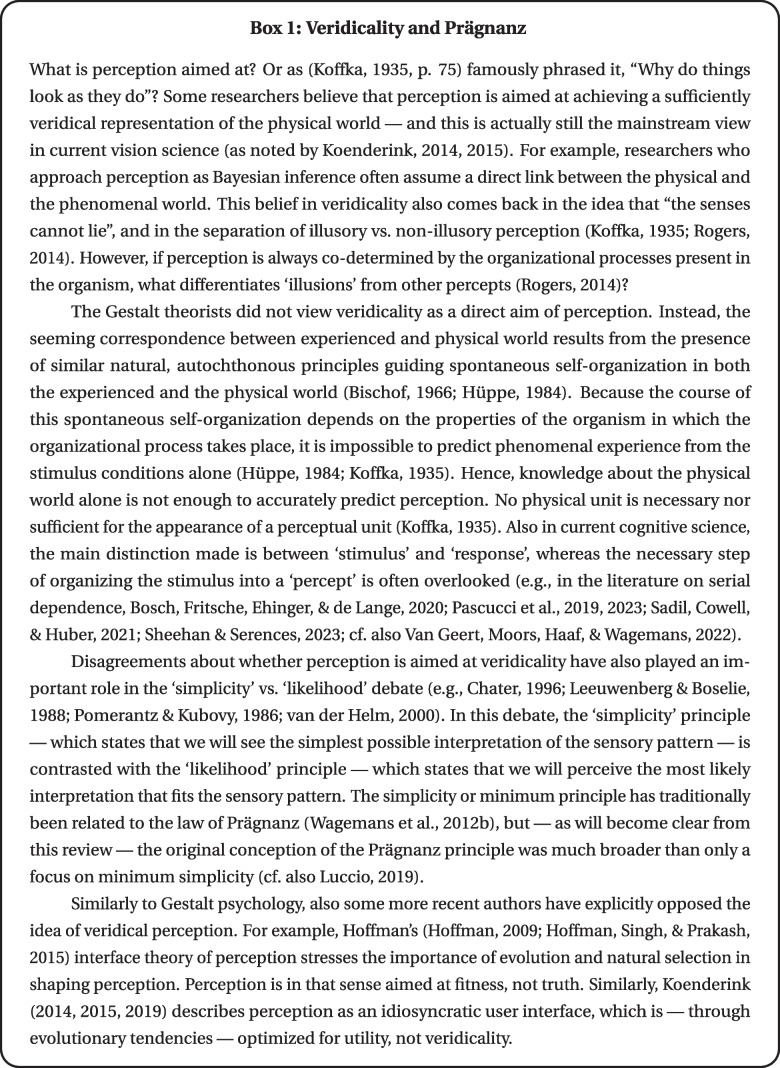


In sum, the tendency towards Prägnanz of a Gestalt indicates a tendency present in every process of psychological organization to come to the organization that — when taking into account the given conditions — has minimal structural energy and is the most clear-cut and simple. Although it concerns a maximal tendency in the direction of high Prägnanz, it does not mean that every organizational process will lead to a simple, clear-cut Gestalt in the absolute sense (Arnheim, 1987; Koffka, 1935; Metzger, 1941). The tendency towards the most prägnant Gestalt should thus always be seen as relative to the prevailing conditions. In addition to a further specification of what a ‘good’, ‘clear-cut’, or ‘simple’ organization entails (cf. Prägnanz as a property), this formulation of the Prägnanz tendency requires clarification of two additional elements: (a) *how* the Prägnanz tendency can be realized; and (b) *which* prevailing *conditions* need to be taken into account.

**Fig. 6:**
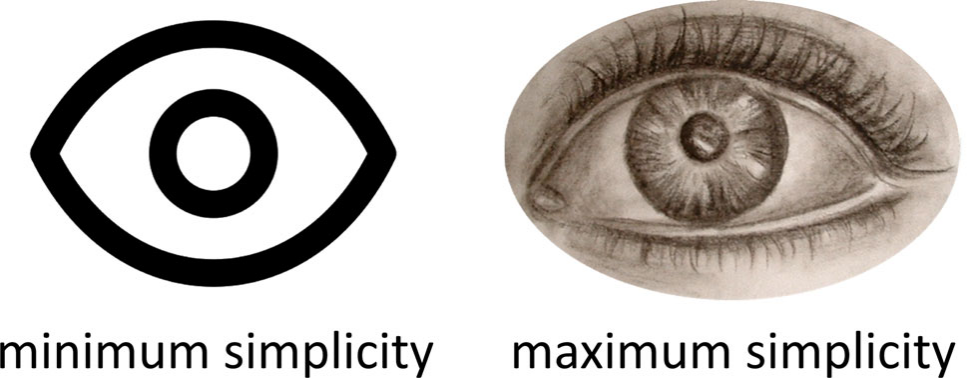
Example of minimum simplicity (i.e., the simplicity of uniformity) and maximum simplicity (i.e., the simplicity of perfect articulation). *Note.* In this Figure, the CC BY licensed eye icon by Shiva from the Noun Project and picture of an eye drawing by Lucky Lynda were used. Figure licensed under CC BY 4.0 by the authors. Retrieved from https://doi.org/10.6084/m9.figshare.21977564

#### *How* can the Prägnanz tendency be realized?

First, *how* can the tendency towards prägnant Gestalts be realized? In psychological organization either *as much* or *as little* will happen as the prevailing conditions permit (Koffka, 1935). Whereas **minimum simplicity** indicates the simplicity of uniformity, **maximum simplicity** indicates the simplicity of perfect articulation (Koffka , 1935; cf. Figure  6). Simplification and complication do not have to be perceived as necessary, mutually-exclusive alternatives, however (Köhler, 1993). To achieve Prägnanz of a perceived whole, some components may need to develop in different directions (Köhler, 1993). Arnheim (1986) viewed simplification and complication as antagonistic but complementary tendencies present in every perceptual event. Whereas tension-reducing tendencies (i.e., simplification, leveling, minimum simplicity) remove unessential details, tension-enhancing tendencies (i.e., complication, sharpening, accentuation, pointing, articulation, maximum simplicity) intensify characteristic features of a Gestalt structure (Arnheim, 1986; Metzger, 1941).^3^ In this way, both simplification and complication can contribute to the Prägnanz of a Gestalt (i.e., they both make the overall organization simpler, more clear-cut, and more unambiguous). Recent work (Prasad & Bainbridge, 2022) concerning the visual Mandela effect in memory is consistent with this idea: some images from popular iconography elicit consistent, specific false memories in the direction of a better Gestalt, regardless of whether it concerns downplaying specific details (i.e., simplification; e.g., a golden instead of a silver leg for C-3PO from the Star Wars franchise) or intensifying characteristic features (i.e., complication; e.g., a black-tipped instead of an almost completely yellow tail for Pikachu from the Pokémon franchise).

Which features will be treated as unessential and which as characteristic? The features that may be noticed refer to differences from a reference used (i.e., a local reference, or an internal reference, cf. ‘Prägnanz steps’). Which of these features will be treated as characteristic will depend on the individual and the context in which the organization is perceived (e.g., Van Geert, Frérart, & Wagemans, 2023).

Importantly, whereas one could equate simplification with literally ‘removing’ features and complication with ‘adding’ features, this does not have to be the case. For example, a square missing one of its four sides may be simplified by adding a sideline, resulting in a complete square. On the other hand, also removing a part of an organization can complicate an organization, e.g., removing a sideline from a full square.

**Fig. 7:**
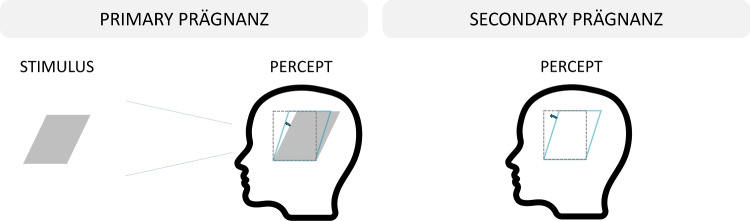
Visual representation of primary and secondary Prägnanz tendencies. The parallelogram represents the incoming stimulus. The shape of the figure is not always perceived veridically. The perceiving individual has a reference distribution of the most prägnant geometric shapes (i.e., Prägnanz steps). In the case of this parallelogram and individual, the rectangle is the reference figure. When perceptually organizing the incoming figure, the perceived figure already deviates from the stimulus (i.e., primary Prägnanz tendency). In this case, the parallelogram is perceived as more rectangular than it actually is (i.e., primary simplification), but under different conditions the parallelogram may be perceived as less rectangular than it actually is (i.e., primary complication). For the perceiver, there is no direct way to be aware of this first deviation. Secondly, the perceiver can also consciously evaluate a perceived organization in relation to a Prägnanz step. In this example, the individual evaluates the shape as ‘almost a rectangle’ (i.e., secondary simplification). Under different conditions, the individual may evaluate the shape as more different from a rectangle than it actually is (i.e., secondary complication). Closeness of the current percept to the internal reference point is directly observable by the perceiver. *Note.* In this Figure, the CC BY licensed head icon by Maxim Kulikov from the Noun Project was used. Figure licensed under CC BY 4.0 by the authors. Retrieved from https://doi.org/10.6084/m9.figshare.21977597

Inspired by Kanizsa’s (1979) distinction between primary perceptual processes (i.e., autochthonous forces leading to the organization of the perceptual field) and secondary perceptual processes (e.g., identification, classification), Hüppe (1984) distinguished primary and secondary Prägnanz (cf. Fig. 7). The **primary Prägnanz** tendency considers the relation between stimulus and phenomenon and leads to deviations from the stimulus to the percept that are not directly noticeable by the observer. Put differently, every phenomenal experience already deviates from the stimulus in the direction of Prägnanz. For example, a parallelogram may be perceived as more rectangular than it actually is (cf. left side of Fig. 7). The **secondary Prägnanz** tendency operates on the phenomenal level and concerns the tendency to evaluate a phenomenon based on its experienced closeness to a prägnant form (i.e., an internal point of comparison, not necessarily phenomenally present). For example, a perceiver may cognitively evaluate the parallelogram as ‘almost a rectangle’ (cf. right side of Fig. 7). Importantly, both Prägnanz tendencies cannot be seen as completely independent: To be able to make statements about secondary Prägnanz, an organized perceptual field (influenced by primary Prägnanz) is preassumed.^4^

#### *Which conditions* influence the course of the Prägnanz tendency?

Second, what do the prevailing conditions entail? As psychological organization takes place in an organism, it is constrained by the conditions outlined by the organism. When it concerns psychophysical processes like human perceptual organization, there are both external and internal conditions to consider (Koffka, 1935). **External conditions** are created within the receptor organs by the proximal stimuli. The *proximal* stimuli, i.e., the excitations to which the light rays coming from the physical object give rise, are in their turn influenced by the *distal* stimuli (i.e., the physical objects), the nature of the light source, the position of the viewer in relation to the distal stimuli and the light source, etc. (Koffka, 1935). **Internal conditions** are related to the structure and state of the human nervous system. Within the internal conditions, more permanent ones (related to the structure of the nervous system, influenced by both inheritance and previous experience)^5^ can be distinguished from more temporary ones (related to, e.g., vigilance, fatigue, needs, attitudes, interests, attentions; Koffka , 1935). One internal condition influencing the tendency towards minimum versus maximum simplicity that Koffka (1935) discussed was the level of vigilance or the energy level of the organism: low activity levels would lead to uniformity and minimum simplicity (i.e., simplification), whereas high activity levels would lead to good articulation (i.e., maximum simplicity, complication; Koffka , 1935).

**Fig. 8:**
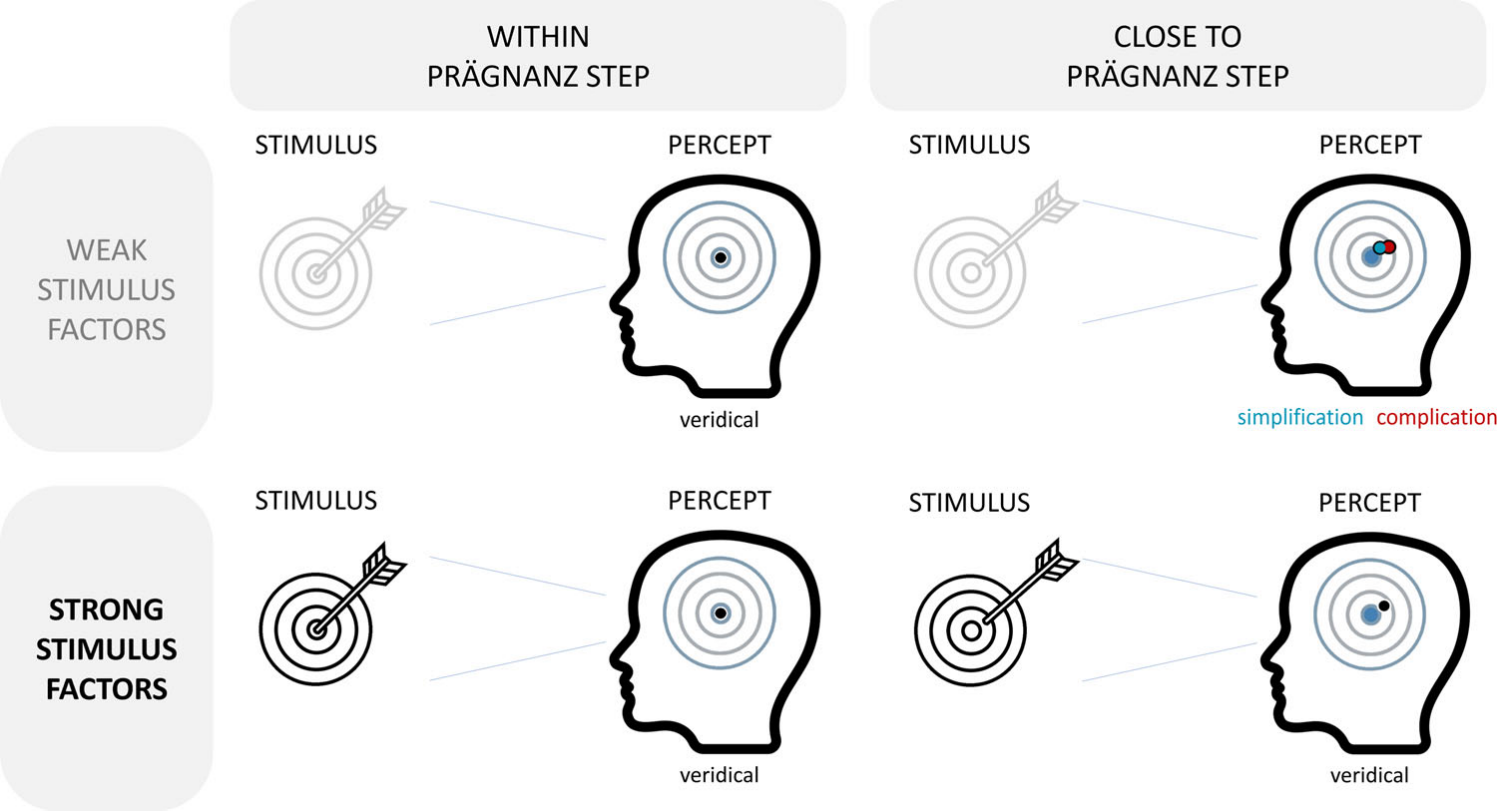
Visual representation of internal and external conditions influencing the Prägnanz tendency. The dartboard represents the incoming stimulus. The location of the arrow hitting the board is not always perceived veridically. The perceiving individual has a reference distribution of the most prägnant locations (i.e., Prägnanz steps). In the case of the dartboard and this individual, the bullseye is the most prägnant region. The circular lines indicated on the board also have some Prägnanz, and the outer circle has a bit more Prägnanz, but less than the bullseye region. When perceiving the location of the arrow on the board, the perceiving individual will take the visual input into account, but also his/her own reference distribution. The tendency towards a better overall organization (i.e., the Prägnanz tendency) will have more room to influence the percept when the external stimulus factors are weak (e.g., because of diminished contrast, short presentation duration, small size). In case the stimulus value (i.e., in this case the location of the arrow on the board) falls within a highly prägnant region, no strong tendency will occur. When the value of the stimulus is close to a prägnant region but falls outside, the Prägnanz tendency will be the largest. Under some circumstances, simplification may occur, pulling the stimulus value closer to the prägnant region (i.e., the bullseye in this case). On the other hand, complication may occur, more clearly differentiating the experienced value from the prägnant region. Which of those two tendencies will occur will depend on the stimulus, person, and context. *Note*. This figure is clearly a simplification of the situation and shows four extreme cases. In any real-life situation, (a) many other stimulus-, person-, and context-related factors are at play (e.g., luminance sensitivity, energy level, stimulus history), (b) stimuli and percepts are multidimensional, which could lead to simplification on one perceptual dimension and complication on another, and (c) the transition between veridicality, simplification, and complication is gradual rather than strictly defined, percepts can be more or less simplified or complicated compared to the actual stimulus value. Furthermore, stimuli that fall exactly within our Prägnanz steps on all important dimensions are very rare compared to stimuli that fall outside of our Prägnanz steps for at least one dimension. The icons in this Figure were adapted from the following CC BY licensed icons from the Noun Project: target by Support Designs and head by Maxim Kulikov. Figure licensed under CC BY 4.0 by the authors. Retrieved from https://doi.org/10.6084/m9.figshare.21977612

The internal and external conditions will serve as two separate organizing forces in perceptual organization. While the internal forces of organization will draw the percept towards the most prägnant organization possible, the external forces will constrain the Prägnanz tendency (Koffka, 1935). When both internal and external forces act in the same direction, very stable organizations should result. In contrast, conflicting internal and external forces will yield a less stable organization (Koffka, 1935).

Although internal forces of organization are present even under conditions of strong external forces (Koffka, 1935), weak external forces (e.g., because of short exposure time, low intensity, small size) will give more room to the internal forces to alter the end Gestalt, producing considerable dislocations which lead to a more stable end state (e.g., Fig. 8). These internal forces can even lead to the addition of new lines if that leads to a better end result (Koffka, 1935). Take note that Koffka also related the laws of organization to the simplicity of the resulting Gestalt, not the simplicity of the process: “the process of organization depends upon the properties of its result” (Koffka , 1935, p. 151).

Although these internal and external conditions influence which Prägnanz tendencies will occur, this conditional dependence does not imply randomness or arbitrariness: it is not the case that ‘anything is possible’ (Wertheimer, 1923; Wertheimer et al., 2012). What it does imply is that stimulus, individual, and context need to be taken into the equation to determine which Prägnanz tendencies will occur under which concrete conditions. Specifically because of these dependencies, the Gestalt psychologists described their Gestalt principles as *ceteris paribus principles*: a Gestalt principle is supposed to hold only within the constraints of the prevailing (internal and external) conditions (Wagemans, 2018).

#### How general is the Prägnanz tendency?

Although this paper focuses on Prägnanz in visual perception, the tendency towards prägnant Gestalts is not at all limited to visual perceptual organization, but was proposed as a *general* tendency present in all forms of psychological organization (e.g., Koffka , 1935; Köhler , 1920; Metzger , 1936/2006). This generality can be interpreted in three different ways. Firstly, the tendency towards prägnant Gestalts is expected to occur and/or has been studied not only in visual perception, but *in all sensory modalities*, as well as *in other, more cognitive areas* of psychological organization (Köhler, 1920; Metzger, 1936/2006), including memory, productive thinking, and problem solving (Kanizsa, 1975; Metzger, 1941; Sorge, 1940; Wertheimer, 1959; Wulf, 1922). Secondly, the Prägnanz tendency is not limited to the perception of very simple proximal stimuli or the use of simple organizational principles like symmetry, but *also occurs under more complex conditions* (Arnheim, 1986; Köhler, 1920). Thirdly, the Prägnanz tendency is expected to occur *in all different species*, and differences between the species are expected to be due to differences in the relative strength of different Gestalt principles rather than to differences in the nature of these principles (Metzger, 1941).

### Prägnanz as a phenomenal property of the resulting organization

In the former part we referred to Prägnanz as a tendency present in every process of psychological organization. However, Prägnanz can also be used to refer to a property of the Gestalt *resulting* from this organizational process. Importantly, **Prägnanz** or **good Gestalt** as a property should be seen as inherently related to the foregoing descriptions of Gestalt and the tendency towards prägnant, simple Gestalts. Nevertheless, it is not the case that every organizational process results in a ‘good’ Gestalt in the absolute sense: it will only result in the best Gestalt possible given the prevailing internal and external conditions. In what follows, we will shed light on the diverse aspects of the Prägnanz concept that have been brought forward to clarify what a ‘good’ or ‘simple’ Gestalt entails.

A first way to characterize Prägnanz as a property is by defining it as **Gestalt height or strength** (Köhler, 1920; von Ehrenfels, 1916, 1932/1937): Strong Gestalts are those Gestalts in which the mutual dependence among the parts is so great that there is no room for any displacement or changes without an influence on all other parts of the whole (Ash, 1995; Köhler, 1920). For example, a rose is a higher Gestalt than a heap of sand (von Ehrenfels, 1916, 1932/1937). The emphasis on strong interdependence between the parts as the defining feature for strong Gestalts may remind you of the original meaning of the Gestalt concept as proposed by the Berlin Gestalt school (i.e., an ensemble of items that mutually support and determine one another; Sundqvist , 2003). (von Ehrenfels, 1932/1937) presented a clear criterion to identify higher Gestalts: “Higher Gestalts are those in which the product of unity of the whole and manifoldness of the parts [*das Produkt von Einheitlichkeit des Ganzen und Mannigfaltigkeit der Teile*] is greater”. Consequently, when one keeps the degree of unity constant, those Gestalts that embrace a greater multiplicity of the parts will be better. Equivalently, for a fixed degree of multiplicity, those Gestalts that more strongly unify this multiplicity will be better (von Ehrenfels , 1916; translated in Smith , 1988). The importance of unity or regularity is also part of Metzger’s (1941) description of Prägnanz: prägnant Gestalts show an outstanding [*ausgezeichnete*] and consequently persistent order (from a purely figural, i.e., non-semantic, perspective). Also more recent literature on how regularity and non-accidental properties increase degree-of-objecthood (e.g., Biederman , 1987; Feldman , 2003; Kubilius et al., 2017; Kubilius et al. , 2014; Strother & Kubovy , 2012; Wagemans , 1992) relates to this characterization of Prägnanz as Gestalt strength.

A second aspect of Prägnanz that Metzger (1941) put forward, next to the aspect of strong figural unity, relates to the etymological meaning of Prägnanz (i.e., being sharply grasping, unambiguous, clear, or distinct; Arnheim , 1975). Metzger (1941) discussed three types of properties of a whole: (a) their structure [*Struktur oder Gefüge*], e.g., straight, round, angular, or symmetrical; (b) their whole quality or texture [*Ganzqualität oder -beschaffenheit*], which is material-related, e.g., transparent, rough, or shiny; and (c) their essence [*Wesen*], e.g., friendly, female, peaceful, or proud.^6^ For each essence [*Wesen*], to the extent that it shows itself in structures [*Gefügen*], there is *a completely specified structure in which the essence is most pure and compelling* (Metzger, 1941, p. 62). This structure is called “ausgezeichnet” or “prägnant”.^7^

For Metzger, both figural order and the pure, compelling embodiment of an essence are essential to understand the full meaning of Prägnanz, and often — if not always — go together (Metzger, 1941).

#### Call for qualititative and quantitative refinement and disambiguation of Prägnanz

Two common critiques on Prägnanz as a phenomenal property are the lack of a sufficiently precise qualitative definition and the lack of a quantitative measure (Metzger, 1966).

##### The lack of a sufficiently precise qualitative definition of Prägnanz

The lack of a sufficiently precise qualitative definition of Prägnanz has often led researchers to point out the ambiguity of the concept as well as to suggest narrower concepts to replace Prägnanz.

Petermann (1931), for example, argued that Prägnanz can only be the start of a formalization as it is much too unclear and undefined. He viewed the Prägnanz tendency as a danger for further progress, as it can serve as a magical solution. Wellek (1959) indicated the dual meaning of Prägnanz as related to clarity and simplicity [***rein figuraler Prägnanz***] as well as meaningfulness and expressiveness [***Sinnprägnanz***]. Although Wellek (1959) believed both aspects of Prägnanz can sometimes covary, he argued that this is predominantly not the case. Therefore, he posits that a distinction between these two meanings is necessary and Prägnanz as a whole is an ambiguous, and hence useless, concept. Kanizsa and Luccio (1986) also pointed to the ambiguity of the Prägnanz concept and distinguished between Prägnanz as excellence, uniqueness, “outstandingness” [***Ausgezeichnetheit***] and as simplicity and stability [***Einfachheit und Stabilität***].

Although these critiques may seem valid at first sight, they may be nuanced or viewed as less destructive based on the foregoing discussion of the origins of the concept. It is true that the Prägnanz tendency can hinder further progress when used as a magical solution (as mentioned by Petermann , 1931), but this was already clear from Wertheimer’s focus on the Prägnanz principle as a general framework to stimulate further investigation into more concrete organizational principles falling under this law and their interactions (Arnheim, 1987; Luchins & Luchins, 1998; Rausch, 1952; Wertheimer, 1924/1999). As Wellek (1959) indicates, Prägnanz is indeed a multifaceted term, and further specifying different aspects of Prägnanz and their relative importance under different conditions should be a continued research endeavor. Although this specification is far from finished, Rausch (1966) undertook a significant effort to clarify Prägnanz as a concept (cf. the section on “Rausch ’s (1966) Prägnanz aspects” below). In the light of its Gestalt psychological context, the seeming ambiguity in the meaning of Prägnanz that Kanizsa and Luccio (1986) indicate (uniqueness vs. simplicity and stability) is maybe less of an ambiguity than it first seems: because organizational processes resulting in a stationary state will tend towards that organization which has minimal energy requirements, the Prägnanz tendency distinguishes stable, simple end Gestalts from other possible states and makes them unique (see also Zimmer , 1991). Gestalt psychologists have deliberately kept these various possibilities open, as part of their view of Prägnanz as a broad, multifaceted concept — a general principle that can take many forms. By distinguishing between diverse facets of the Prägnanz concept, the so-called ambiguity or vagueness can be avoided, and empirical research can investigate under which conditions the different manifestations occur. Conceptual refinement is thus a necessary condition for empirical progress, which is an important motivation for this review detailing the nuances in the original meanings of Prägnanz.

##### The lack of a quantitative measure for Prägnanz

Some researchers aimed to replace the qualitative Prägnanz concept with a narrower, more easily quantifiable concept. For example, Hochberg (1968) suggested that an objective definition of ‘simplicity’ is needed if we want to be able to predict how an ambiguous image will be perceived. Also, he compared the lack of a quantitative measure for simplicity with the lack of a quantitative way to determine likelihood (i.e., how do we know that what we perceive is the most likely interpretation?; cf. also the ‘simplicity’-‘likelihood’ debate, e.g., Chater , 1996; Leeuwenberg & Boselie , 1988; Pomerantz & Kubovy , 1986; van der Helm , 2000).

Goldmeier (1937, 1972) reduced Prägnanz to the concept of **singularity**: singular (i.e., unique) qualities are those qualities that are very sensitive to change, and are contrasted with qualities that have a range character and consequently are insensitive to change. Yet Arnheim (1987) called the concept of singularity misleading: things can be unique for many reasons that are totally unrelated to Prägnanz. Singularity is only a secondary consequence of the purity of prägnant forms (Arnheim, 1987).

In the light of information theoretical approaches, Prägnanz has been equated with high **internal redundancy** (i.e., low information content; Attneave , 1954; Hochberg & McAlister , 1953)^8^. Hochberg and McAlister (1953) proposed to search for parallels of the qualitatively and subjectively formulated Gestalt principles of perceptual organization by analyzing the objective properties of the stimulus constellation. As an objective definition of perceptual “goodness” they posited the frequency of occurrence of, or the relative time span devoted to, each perceptual organization that a stimulus may elicit. They hypothesized that the organization requiring the least information to be specified, will be the most likely to be perceived. To approximate figural goodness in this way, it was deemed important (a) to empirically determine the dimensions on which information needs to be scored, and (b) to demonstrate a correlation between the frequency with which different organizations of a stimulus constellation occur and the calculated information scores for a large set of stimuli.

Attneave (1954) operationalized Prägnanz as internal redundancy: the most prägnant figure will be the one with the highest degree of internal redundancy^9^. In his view, the different grouping principles specified in Gestalt psychology all stimulate redundancy.

Garner (1974) proposed subset size to be the critical aspect of redundancy in relation to pattern goodness: good patterns are part of small subsets and this relates to high internal redundancy. This subset size can be compared to the size of the total set, which contains all stimuli that can be produced given a specific set of dimensions and levels (Garner, 1974). Whereas in some cases the actual subset size may be known, an inferred subset can be produced for any single stimulus. Garner (1974) describes evidence for the beneficial effects of pattern goodness as indicated by inferred subset size on several information processing tasks, including perceptual discrimination, recognition memory, reproduction memory, and verbal encoding of stimulus patterns (Attneave, 1955; Checkosky & Whitlock, 1973; Clement, 1964; Clement & Varnadoe, 1967; Glanzer & Clark, 1963; Pomerantz, 1977; Pomerantz & Garner, 1973). One such finding is that good patterns are encoded more rapidly (Garner, 1974; Pomerantz, 1977). Often sets of dot patterns are used in these tasks, with the number of reflections and 90^∘^ rotations that lead to different patterns than the given one determining subset size.

According to Palmer (1982), good figures are those that have greater transformational invariance (i.e., contain more symmetries). More specifically, Palmer (1991) extends Garner’s (1974) idea of reflection and rotation subsets and proposes a figure to be good when there are more local and global transformations (e.g., rotation, reflection) that leave the figure unchanged.

**Fig. 9:**
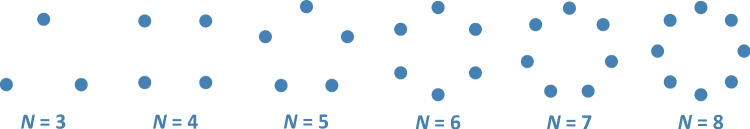
Dot patterns suggesting regular polygon shapes. *N* indicates the structural complexity of the pattern as defined in Koenderink et al. (2018). These figures were created using the OCTA toolbox (Van Geert, Bossens, & Wagemans, 2023), with the intention to resemble Figure 7 from Koenderink et al. (2018). Figure licensed under CC BY 4.0 by the authors. Retrieved from https://doi.org/10.6084/m9.figshare.21977645

In structural information theory (Leeuwenberg & van der Helm, 2012), another approach to quantifying Prägnanz and the Prägnanz principle, the stimulus organization that we will perceive is expected to be the one containing the least structural information (i.e., simplicity or descriptive minimum principle). Three basic types of regularity are distinguished: iteration (e.g., AAAA), symmetry (e.g., ABBA), and alternation (e.g., ABCB). The stimulus organization can involve hierarchically organized levels, of which the highest hierarchical level (i.e., the ‘superstructure’) will determine the perceived unity (Leeuwenberg & van der Helm, 1991). Structural information theory is mainly meant for intra-stimulus comparisons of different possible organizations of the same stimulus (Leeuwenberg & van der Helm, 2012; van der Helm, 2017; van Lier, van der Helm, & Leeuwenberg, 1994): for each stimulus, the most structurally simple interpretation will be perceived. When different stimuli are compared, the simplest descriptions for each of the stimuli are used and figural goodness is used as a criterion (Leeuwenberg & van der Helm, 2012). In this context, figural goodness is explicitly distinguished from simplicity and is defined as the detectability of (or weight of evidence for) a regularity in a stimulus.

Importantly, a reduction of the Prägnanz concept to internal redundancy or inferred subset size completely focuses on stimulus-related aspects and leaves out any influences of the observer on Prägnanz (see also Koenderink et al., 2018). Hochberg (2003) later admitted that this purely stimulus-based approach was too simplistic: for example, attention and meaningfulness or familiarity will also influence the organization that we perceive (e.g., Peterson & Gibson , 1994). Metzger (1975) pointed to the specificity of information theory-based research’s results when treating Prägnanz as a negative entropy or redundancy measure, and strongly doubted the generalizability of these as measures for Prägnanz.

Whereas structural information theory focuses on how stimuli are perceived rather than on aspects of the stimuli themselves, the theory also leaves out any influences of the observer on what the most prägnant organization will be. Hence, this theory also does not provide a satisfying formalization of Prägnanz.

In many of these quantifications of Prägnanz, the focus has been on ‘simplicity’ rather than the more meaning- or complexity-related aspects of the Prägnanz concept (Hüppe , 1984; Koenderink et al. , 2018; Luccio , 2019; cf. the section on “Rausch’s (1966) Prägnanz aspects” below). For example, the complexity of the individual components in the stimulus constellation is not taken into account. This is possibly a consequence of the type of stimulus materials used in most studies: in dot lattices for example, element richness, expressiveness, and meaningfulness do not play a role (Hüppe, 1984).

Koenderink et al. (2018) investigated whether a quantification of the Prägnanz concept is possible at all, or more specifically, whether there are aspects or interpretations of the concept that can be quantified. They deviate from the tendency to focus on the stimulus structure alone, and also consider the observer as an important determinant of Prägnanz. If a measure for Prägnanz is to be found, it needs to take into account both the structural complexity^10^ bottleneck of visual systems (i.e., an upper limit to the complexity that can be processed by the organism) and the relevance to the organism’s biological fitness (Koenderink et al., 2018). As an example of Gestalts high in Prägnanz, the releasers or sign stimuli discussed in ethology are mentioned (i.e., stimulus constellations that trigger a fixed behavioral pattern in a particular species). Koenderink et al. (2018) state that in humans, the structural complexity bottleneck might be enough to approximate Prägnanz with some generality. When the structural complexity of a stimulus exceeds the level of structural complexity that the organism can process, the stimulus will no longer be recognized as a “picture”, but rather as “featureless” or “just noise” (Koenderink et al., 2018). Within the capacity limits of the visual system, the more structurally complex stimulus will be experienced as more prägnant. This is because more complex patterns (that stay within the capacity limits of the organism) will be experienced as more unique and hence raise the odds of detection, while they are still ‘simple enough’ to ensure automatic detection (Koenderink et al., 2018). As a concrete example: given that the maximum structural complexity that can be processed by the organism is equal to 4 (*M* = 4), patterns with a complexity of 4 (*N* = 4) will be experienced as most prägnant (*P* = *N*/*M* = 1). For patterns that exceed the structural complexity bottleneck of the organism (*N* > 4), Prägnanz will drop abruptly (cf. Fig. 9).

**Fig. 10:**
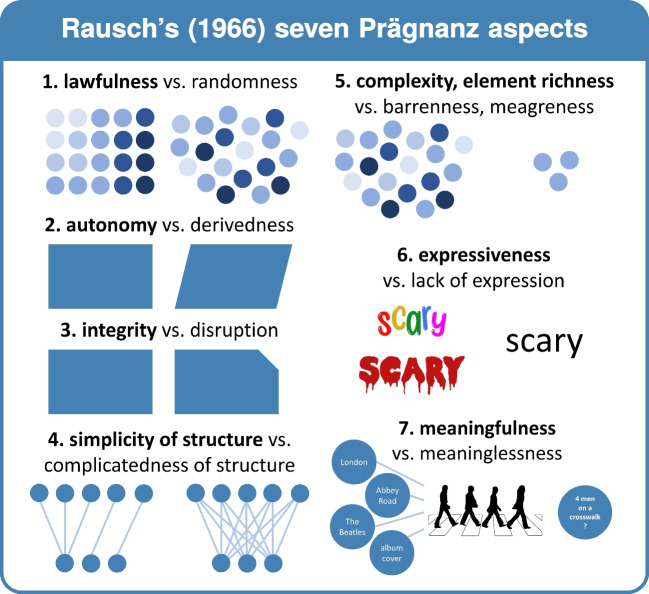
Illustration of the seven groups of Prägnanz aspects as defined by Rausch (1966). The Abbey Road icon was adapted from the CC BY licensed AbbeyRoad icon by Lia Thompson from the Noun Project. Figure licensed under CC BY 4.0 by the authors. Retrieved from https://doi.org/10.6084/m9.figshare.21977693

In general, although several researchers have tried to replace Prägnanz with narrower, more easily quantifiable concepts, the Prägnanz concept – as originally conceived, not as later interpreted – cannot be replaced (Metzger, 1941). Its multifaceted nature certainly needs further specification and study, but this multifacetedness is exactly what is essential to make Prägnanz a viable concept. This also means that any interpretation of Prägnanz as purely figural, not taking into account the individual and context in question, will eventually fail. For example, when visually grouping a set of known objects, essential properties like purpose of use will more often play a determining role than structural (e.g., color, size, shape) or material properties, and complementary objects will preferably be grouped, rather than objects with the same function (Metzger, 1941).

Each of the mentioned quantifications of Prägnanz has led to valuable contributions. Importantly, it is not bad to try to quantify Prägnanz in a specific context for a specific set of stimuli, rather to the contrary. Nevertheless, these measures are too preliminary and too stimulus- and context-specific to choose one quantification and thereby replace the overall concept of Prägnanz.

None of the listed critiques counters the essence of the concept (Metzger, 1966), nor do they imply that the scope of Prägnanz’s application should be limited (Rausch, 1952). It is only important to recognize that (a) there is a need for further concretisation of Prägnanz, and (b) besides the general principle, more detailed, possibly quantitative, specifications of individual cases are also relevant (Rausch, 1952). Rausch (1966) made a laudable effort to qualitatively clarify different aspects of Prägnanz, and also proposed some early quantitative indicators of Prägnanz.

#### Rausch’s (1966) Prägnanz aspects (cf. Fig. 10)

As Rausch (1966) posits, Prägnanz is a highly complex concept, which makes it necessary to further specify its different aspects. Rausch (1966) distinguished seven Prägnanz aspects, and although these aspects are still complex in themselves (allowing for several different expressions of the same aspect), and the naming of these aspects is somewhat arbitrary (as a term is not always available to capture the full commonality within that aspect), these aspects may help to clarify the diversity of Prägnanz in its original meaning. Rather than only mentioning the overarching Prägnanz concept, Rausch (1966) advises future researchers to also specify which aspect of Prägnanz one refers to. The first four Prägnanz aspects highlight aspects of lawfulness or regularity (i.e., order, unity), the other three focus on aspects of ‘fullness’, complexity, or multiplicity [*Fülleaspekte*]. Five of the seven specify purely form-related aspects of Prägnanz, whereas the two last aspects are more content-related. Dependent on the phenomenon under consideration, one can use a system of 3, 4, 5, 6, or 7 Prägnanz aspects to evaluate its Prägnanz (Rausch, 1966). In what follows, Rausch’s (1966) discussion of Prägnanz aspects is translated and summarized.^11^

##### 1. Lawfulness vs. randomness

This first Prägnanz aspect reflects both the clarity of ***unity*** or degree of unity of a complex or Gestalt (or the clarity of the existence of a Gestalt quality) and its ***lawfulness*** or regularity (as opposed to randomness). Importantly, unity cannot only be reached by uniformity or homogeneity, but also by other types of structuring. Uniformity thus only presents a special case. As the experienced lawfulness (i.e., regularity, order) of a complex indicates or co-determines its experienced unity (and lawfulness and unity thus are not completely independent of each other), one can characterize lawfulness (as opposed to randomness) as the decisive factor for this first Prägnanz aspect. Whereas this aspect can be binary in experience (i.e., a complex is either ordered or not), it is also possible to speak of a degree of lawfulness. The other Prägnanz aspects depend on the presence of at least some form of lawfulness or regularity. As a reminder, it is important to take into account that it concerns the perceived lawfulness of a *phenomenal experience*. That means that whereas for one individual, a relative complex stimulus constellation can lead to a phenomenal experience with a random character, another individual or different conditions can yield a lawful phenomenal experience based on the same stimulus.

##### 2. Autonomy vs. derivedness

The second Prägnanz aspect distinguishes phenomena (or phenomenal qualities) that are ***autonomous*** rather than derived, in a binary fashion. For example, one could say that a parallelogram is derived compared to a rectangle (which is seen as autonomous). Similarly, obtuse and sharp angles can be seen as derived from the autonomous right angle. As a phenomenon can be autonomous in one dimension and derived in another, it is important to specify the dimension under evaluation. Derivations can occur in form or shape, but also in position (i.e., location and orientation). The relation between autonomous and derived is asymmetrical and non-reversible: whereas the parallelogram can be seen as derived from the rectangle, the rectangle cannot be seen as derived from the parallelogram. Although Rausch (1966) first posits this Prägnanz aspect to be dichotomous (i.e., a phenomenon is either autonomous or derived), he also acknowledges that a more gradual interpretation of autonomy is possible. For example,^12^ one could say that a rectangle is derived from a square, and then a rectangle would be more autonomous than a parallelogram, but less autonomous than a square (see also Feldman , 2000; Hendrickx & Wagemans , 1999; Leyton , 1992; Sablé-Meyer et al. , 2021; Wagemans et al. , 1994).

##### 3. Integrity vs. disturbedness

***Integrity*** or completeness rather than disturbedness^13^ forms the third (dichotomous) Prägnanz aspect put forward by Rausch (1966). This disturbedness can be related to the stimulus constellation underlying the phenomenon as a whole or more locally, and can manifest in diverse ways: something can be missing, superfluous, or different; something can be ‘not yet complete’ or ‘not complete anymore’ (cf. also Spröte et al., 2016). In each of these cases, a complete version indicating how the complex should look serves as a reference. Similar as the relation between autonomous and derived in the second Prägnanz aspect, the relationship between distorted and complete is asymmetric. Although Rausch (1966) first posits this Prägnanz aspect as strictly dichotomous (i.e., a phenomenon is experienced as either complete or disturbed), he later acknowledges the possibility of a more gradual interpretation. One could say that a certain phenomenon or phenomenal quality is more or less disturbed, for example the extent to which an alignment of elements deviates from a straight line^14^ (Claessens & Wagemans, 2008; Strother & Kubovy, 2006).

How can one distinguish between derivedness and disturbedness? This may have to do with the distance from the prägnant form. If a parallelogram is ‘almost a rectangle’, or an angle is ‘almost right’, it will be perceived as distorted. If the phenomenon is far away from the prägnant form, it may be perceived as derived. The precise distinction between the two is not always clear. In that case, one can talk about a *deviation* from a Prägnanz step (i.e., the complete or autonomous form of the complex, cf. the section on “Prägnanzstufen” below) to refer to either derivedness or disturbedness.

##### 4. Simplicity of structure vs. complicatedness of structure

Although it is easy to confuse this fourth Prägnanz aspect of ***simplicity of structure*** with the first (i.e., lawfulness or regularity), it can be distinguished from it. More specifically, regularity can be simple or complicated. Put differently: To be prägnant, an organization should at least contain some form of regularity. Within prägnant organizations, there is a tendency towards simple regularities. Simplicity of structure can be seen as a binary concept or as a continuum.

##### 5. Complexity (part or element richness) vs. sparseness

Given a fixed level of regularity, a phenomenon will be experienced as more prägnant when it contains a greater number or a greater diversity of components, i.e., when it is more complex (as opposed to sparse). Importantly, ***complexity*** is different from the above mentioned complicatedness: whereas complicatedness relates to the entanglement and intricacy of a regularity or structure, complexity relates to the comprehensiveness and encompassingness of a phenomenon as indicated by the multiplicity and diversity of its parts. This fifth Prägnanz aspect can be used as a dichotomy or as a graded property. As all other Prägnanz aspects, complexity is meant as a phenomenal property, not as a property of the stimulus constellation. Individual and contextual differences can thus occur.

##### 6. Expressiveness vs. weakness of expression

A phenomenon is experienced as more prägnant, the more expressive the phenomenon is. With this sixth Prägnanz aspect, Rausch (1966) goes beyond purely structural properties of a phenomenon. ***Expressiveness*** relates to Metzger’s (1941) definition of Prägnanz as a pure embodiment of a nature or essence. For this Prägnanz aspect to be usable, the range of variability between different phenomena to be compared should be limited.

##### 7. Meaningfulness vs. meaninglessness

The seventh Prägnanz aspect of ***meaningfulness*** deals with being able to connect a phenomenal experience with earlier acquired knowledge. For example, when perceiving an acquaintance, we can connect the face of this person with where he lives, which profession he has, which opinions he holds, etc. The richer the connections with previous knowledge, the more meaningful the experience of the phenomenon. Unlike expressiveness, which is inherently and from the start related to a structure or organization, meaning is added later. Similarly to the sixth Prägnanz aspect (i.e., expressiveness), meaningfulness can in practice only be used as a Prägnanz aspect when the range of variability between different phenomena to be compared is constrained. When Wellek (1959) distinguished purely figural Prägnanz from Sinnprägnanz, the latter referred to both expressiveness and meaningfulness.

#### Implications of Rausch’s (1966) Prägnanz aspects for Prägnanz tendencies

As Rausch (1966) indicates, the further specification of Prägnanz in several Prägnanz aspects can also help to distinguish several Prägnanz tendencies. Oftentimes the Prägnanz tendency refers to a general tendency towards lawfulness and unity (i.e., the first Prägnanz aspect). In other cases, the Prägnanz tendency may refer to a tendency towards autonomy, towards completeness, or towards simple structure. Under weak stimulus conditions, it may be the tendency from distorted to complete that is most prominent. The tendency from derived to autonomous may occur in a more conscious manner. Both the tendency towards autonomy and towards completeness are tendencies towards a latent reference (i.e., the autonomous or complete ‘original’ of the phenomenal experience). Further research can clarify the strength of each of these Prägnanz tendencies under different conditions.

#### Rausch’s (1966) quantitative indicators of Prägnanz

Rausch (1966) specified three quantitative characteristics or features (not ‘measures’) of Prägnanz. Although these quantitative concepts are defined here, they are in need for extension, as are the seven Prägnanz aspects mentioned (Rausch, 1966).

##### Density of Prägnanz steps (Prägnanzstufendichte; D)

As a characteristic of an individual, the quantitative indicator of *Prägnanz step density* concerns the degree of differentiation present when distinguishing and using prägnant steps (cf. the section on “Prägnanzstufen” below) in a certain domain. The higher the number of separate, clearly distinguishable ranges on a certain dimension, the higher the density of prägnant steps. It can be interpreted either as a temporary state or as a more permanent trait of an individual.

##### Prägnanz strength (Prägnanzstarke; S)

*Prägnanz strength* takes into account three to seven Prägnanz aspects in a dichotomous fashion, and indicates how many of those Prägnanz aspects are present in a phenomenon. The feature thus has a value between 0 and N, the number of N being the number of Prägnanz aspects taken into account. For example, a rectangle is prägnant on all three first Prägnanz aspects (S = 3), whereas a parallelogram is lawful and complete, but derived (S = 2).

##### Autonomy index (Eigenständigkeitsindex; J)

The *autonomy index* Rausch (1966) proposed only takes the second Prägnanz aspect into account, and deals with the question of how many important properties or dimensions of a phenomenon are autonomous or derived. For example, when taking equal width, straightness, and orthogonality into account as properties (cf. Rausch , 1952), a rectangle is autonomous on all three (J = 3), whereas a parallelogram lacks orthogonality and is, in that sense, derived (J = 2).

### Prägnanzstufen

Ideas linked to the concept of **Prägnanz steps** [*Prägnanzstufen*] were present already in Wertheimer’s (1912) article on numerical thinking in aboriginal people, *“Über das Denken der Naturvölker. I. Zahlen und Zahlgebilde”* (Hüppe, 1984). There, Wertheimer (1912) mentioned *ausgezeichneten Anzahlen* (§10, p. 337–340), literally translated as “out-standing”, unique numbers. Some of these unique numbers become apparent from how they are named (e.g., in Andamanese, 10 = orduru = “all”). They are also used for the formation of higher numbers and in the rounding of prices. In addition, numbers close to them are expressed in terms of these unique numbers, which serve as a reference point (e.g., in Ralik Rakater: 8 = “take two away”, where 10 is the reference point). These unique numbers serve as a first example of *Prägnanzstufen* (i.e., *Prägnanz* steps or reference regions), a concept elaborated further in Wertheimer’s (1923) article *“Untersuchungen zur Lehre von der Gestalt. II.”*.

It was only in 1923 that Wertheimer first discussed *Prägnanz* as a Gestalt principle and its relation to Prägnanz steps in more detail, although he conducted the research during his period in Frankfurt (1911-1914, Wertheimer , 1923; translated in Wertheimer et al. , 2012). He used series of dots as well as angles of varying degrees to illustrate that in between distinctive regions (in which there is a clear, “winning” grouping or organization available), there are often intermediate series that are “not unequivocal to the same degree, not quite as salient [*prägnant*], ‘less definite’ in their character, less pronounced, and often more easily seen in terms of one grouping or the other” (Wertheimer, 1923; Wertheimer et al., 2012). The *Prägnanz* principle thus entails that if one varies a component (e.g., the location of a dot in between two other dots) in systematic, physically equidistant steps, the resulting psychological impressions will not be equidistant; the progression will be discontinuous as particular Prägnanz steps (i.e., *Prägnanzstufen*) occur, that each have their own range (of influence).

Between different Prägnanz steps, three situations can occur. *In between* the ranges of influence of different Prägnanz steps, either neutral transition areas can be present (in which percepts are indifferent or meaningless) or ambiguous percepts may occur that fluctuate between two Prägnanz ranges (Rausch, 1966). *Within* the ranges of influence of the Prägnanz steps, less prägnant forms will be experienced as related to the prägnant forms, as somewhat “poorer”, incomplete, disturbed versions of them (Rausch, 1966; Wertheimer, 1923; Wertheimer et al., 2012). For example, an angle of 93^∘^ may look like a right angle, but not completely (Rausch, 1966). Put differently, less prägnant forms that are close to Prägnanz steps (i.e., in their range of influence) are *evaluated* in relation to these prägnant forms, but can be *perceptually* discriminated from them (cf. also the distinction between primary and secondary Prägnanz tendencies described by Hüppe , 1984).

Prägnanz steps thus serve a double function. On the one hand, assimilation (i.e., attraction, simplification) to the Prägnanz steps may occur, especially when the external conditions are weak (i.e., limited visibility, due to, e.g., brief presentation, low contrast, or small size; Köhler , 1920; Stadler et al., 1979; Wertheimer , 1923; cf. also Van Geert & Wagemans , 2023)^15^. On the other hand, Prägnanz steps can increase sensitivity to change in their vicinity: when viewing conditions are less limited, they can increase the ability to notice small deviations from a Prägnanz step (Goldmeier, 1937, 1982). In that sense, Prägnanz steps can support both robustness and sensitivity in visual experience, via simplification and complication, respectively (cf. the section on “How can the Prägnanz tendency be realized?” above).

Furthermore, there is an asymmetric relationship between less prägnant forms and the Prägnanz steps they are related to: “the bad Gestalt looks similar to the out-standing one, but not the other way around” (Metzger , 1941, p. 63; cf. also Goldmeier , 1937, 1982). In addition, a hierarchy of Prägnanz steps can exist: for example, the right angle will be a better Gestalt than the sharp or obtuse angles (following the three first Prägnanz aspects), although the sharp and obtuse angles can also be seen as Prägnanz steps (Rausch, 1966).

*Prägnanzstufen* or Prägnanz steps are thus exemplars of good Gestalts, as they embody a particular essence purely (Rausch, 1966). The number of Prägnanz steps can increase with experience or time, as new intermediate steps may develop, with the new steps forming as embodiments of special subtypes or subclasses of an essence in areas that are only meaningless intermediate areas for less sensitive individuals (Metzger, 1941; Wertheimer, 1923; Wertheimer et al., 2012). Individuals may not only differ in the number of Prägnanz steps they have on a dimension, but also in the width of their Prägnanz steps: more sensitive individuals may have narrower and more sharply centered Prägnanz steps (Metzger, 1941). These Prägnanz steps were primarily defined on purely quantitative dimensions, but Wertheimer (1923; Wertheimer et al. , 2012) noted that something similar occurs in the purely qualitative domain. For example, the system of Prägnanz steps concerning animal shapes for a zoologist, or concerning colors for a painter, will contain more, but also narrower, Prägnanz steps than the corresponding systems in children (Metzger, 1941). More recently, also the ‘geons’ (i.e., geometrical ions) that are a crucial part of Biederman’s (1987) recognition-by-components (RBC) theory could be seen as reference shapes (i.e., Prägnanz steps) for recognizing object parts. Of course, 3D shapes could be differentiated better than the 2-fold or 3-fold distinctions on the 4 dimensions underlying the 36 components (2x2x3x3) that Biederman proposed as building blocks, but as building blocks for rapid and automatic object recognition, these would be sufficient according to RBC theory.

Rausch (1952) distinguished **Prägnanz height** (or dimensionality) from Prägnanz steps (or the Prägnanz function): Prägnanz height is used when multiple (objective) variables play a role, Prägnanz steps when only one (objective) property is varied. Although we call it Prägnanz ‘steps’, these do not refer to a stepwise function; we should interpret them as values from an objective variable (Rausch, 1952). In other words, we can look at a variable dimension as having a Prägnanz function across its domain: some regions have higher Prägnanz than others (Rausch, 1966), and it clearly concerns a gradual concept.

For a single objective domain, sometimes Prägnanz steps will be present and sometimes more homogeneous perception will arise. For example, when we speak of the time as ‘five minutes before nine’, we use nine o’clock as a reference point. When we however use a concurrently present local reference, like the small hand in a clock as comparison for the big hand, Prägnanz steps can be absent (Rausch, 1966). Relatedly, if a stimulus is presented as part of an ordered series, the factor of objective set or setting [*Einstellung*] comes into play, and Prägnanz steps will no longer be the only factor determining the resulting organization (Wertheimer, 1923).

#### Prägnanz steps in the work of Eleanor Rosch

The work of Eleanor Rosch on perceptual and cognitive reference points (e.g., Rosch , 1975) also builds on the idea of prägnant steps. Rosch (1975) viewed stimuli as ‘reference points’ when other stimuli are seen ‘in relation to’ them. She indicated that categories are often not clearly delineated, but rather built around prototypes (i.e., clearest cases, best examples). These prototypes exemplify the ‘core meaning’ of the category (cf. Prägnanz as pure embodiment of an essence). These core meanings around which categories build are in no sense arbitrary, but are given by the human perceptual system: they are more perceptually salient than other exemplars, hence ‘natural’ prototypes (Rosch, 1973).

We also do not need clear category boundaries to be able to judge the degree of prototypicality of an exemplar, but rather use clear cases as a comparison, an insight she attributed to Wittgenstein (Rosch, 1978). Non-prototype category members trend towards the prototype to a certain extent, and this may lead to systematic asymmetries in, for example, perceived similarity (Rosch, 1975).

Importantly, just as in the Gestalt view, Rosch (1978) does not interpret ‘prototypes’ as one single value on a dimension, but rather emphasizes the gradualness of prototypicality. In her view, a structure is more prototypical when it has more attributes in common with other members of the category, *and* when it has fewer attributes in common with members of contrasting categories (Rosch, 1978). Which attributes will be perceived is partially dependent on the functional needs of the organism (Rosch, 1978). Degree of protoypicality also correlated with beneficial effects on reaction time, speed of learning, etc. (Rosch, 1978).

Although Rosch (1975) was aware of Wertheimer’s work on Prägnanzstufen – she described it as the idea that there are certain ‘ideal types’ which serve as anchoring points for perception (Rosch, 1975), and her husband was the son of Fritz Heider, a psychologist working in the Gestalt psychological tradition – she never discussed the Gestalt ideas in detail (Bock & Pfeiffer, 1987; Hüppe, 1984).

### Aesthetics and Gestalt

When we psychologically organize incoming stimuli, we can *not only* describe or classify the experienced organization in a purely structural or semantic sense, we can also evaluate our aesthetic experience of this organization. Perceptual processing of the input is necessary to be able to aesthetically evaluate our percept, therefore the close relation between perception and aesthetics cannot be neglected.

**Fig. 11:**
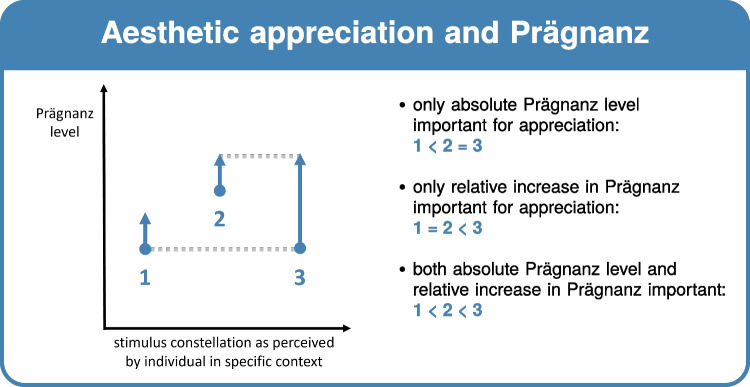
Potential relations between aesthetic appreciation and Prägnanz. Figure licensed under CC BY 4.0 by the authors. Retrieved from https://doi.org/10.6084/m9.figshare.21977762

Goodness of Gestalt has been tied to aesthetic appreciation since the beginnings of Gestalt psychology. von Ehrenfels (1922, p. 50) made the relation clear as follows: “what we call beauty is nothing else than Gestalt height”. According to Arnheim (1986, p. 823), Wertheimer spoke of ‘good’ Gestalts because of the cognitive and aesthetic improvement they bring about. Koffka indicated that violations of organizational principles like good continuation and good shape due to external conditions are felt as violations because they “hurt our sense of beauty” (Koffka , 1935, p. 175). Moreover, those stimulus constellations that are most in agreement with the organizational principles underlying our perception will be judged as most beautiful (Eysenck, 1942).

Aesthetics has a clear relation to the Prägnanz *tendency* as well. Metzger (1941) indicated that true artists will go beyond their models in the direction of Prägnanz, which means that they will come to a structure that more purely and compellingly specifies the essence of content of the artwork. To make the essence clearer, they can use simplification and/or complication strategies. Also in art, these tension-reducing and tension-promoting tendencies are always concurrently present to a certain extent (Arnheim, 1975). In his late works, Piet Mondrian, for example, used rectangularity and primary colors, which served to simplify, but the irregular spacing was a complication, which served to make his work more dynamic (Arnheim, 1975).

The same tendencies — order and complexity, unity and variety, integration and differentiation, simplification and articulation — are thus at play as determinants of aesthetic appreciation and those of good Gestalt or Prägnanz in visual perception (Eysenck, 1942). In perception, we are constrained by the external conditions in our tendency towards Prägnanz, and these external conditions will typically not allow for highly balanced and symmetric percepts. When the external conditions do allow balance and symmetry, however, it will be perceived (Koffka, 1940). In that sense, perception is artistic (Koffka, 1940). Artworks on the other hand are made with balance and symmetry in mind, they are made to serve as as a source of stimulation that results in the perception of a good Gestalt (Koffka, 1940; Smith, 1988).

The artist will thus trigger tendencies in the observer to experience order, but different types of Prägnanz tendencies may be triggered and artists may differ in the type of Prägnanz they aim to maximize (Smith, 1988). Exactly the multidimensionality of Prägnanz makes the work of artists extremely difficult, as it is unclear how different Prägnanz tendencies will interact when concurrently present (Smith, 1988). Knowing about the diverse aspects to Prägnanz might support artists in finding dimensions of aesthetically relevant structure in both their artworks and the reactions to their artworks, without dictating how an artwork should look (Smith, 1988).

In our view, a minimum level of unity or order may be a prerequisite for aesthetic appreciation as it is for Prägnanz, but aesthetic appreciation is expected to arise together with a conscious increase in Prägnanz. This increase may be seen as a comparison between organizations (i.e., one organization is experienced as more prägnant than the other), or as an improvement in the experienced organization keeping the stimulus constant. For example, by extended looking, repeated viewing, and/or expertise, one may notice some kind of higher-order relationship between elements that were initially perceived to be disconnected or in arbitrary positions. The percept then becomes increasingly better organized even though all stimulus elements remain the same. Following this idea, aesthetic appreciation will be higher when a stronger Prägnanz *tendency* is experienced, which can be the result of an increase in any of the mentioned aspects of Prägnanz (either related to order or complexity). It is important to note, however, that increased complexity will only lead to increased appreciation up to the point the observer can still grasp the resulting organization (cf. above).

This view (see also Van Geert & Wagemans , 2020) is similar to other accounts of aesthetic appreciation, including the predictive processing accounts of Van de Cruys and Wagemans (2011) and Chetverikov and Kristjánsson (2016) as well as the focus on pleasure by insights into Gestalt proposed by Muth and Carbon (2013, 2016; Muth et al. 2013). An alternative view, more in line with the interpretation by von Ehrenfels (1922) and Eysenck (1942), is that aesthetic appreciation could be based on the absolute level of Prägnanz experienced. The absolute level of Prägnanz (i.e., Prägnanz *height*) does not necessarily relate to the strength of the experienced Prägnanz *tendency* (i.e., relative increase in Prägnanz height; see Fig. 11). Nevertheless, both views may act complementarily as well. This combination of views is in line with the pleasure-interest model of aesthetic liking proposed by Graf and Landwehr (2015, 2017).

## Discussion and conclusion

By looking back at the history of Prägnanz, we aimed to give a more detailed overview of the different uses and interpretations of the concept than is typically done. We distinguished four main uses: (a) Prägnanz as a tendency present in each process of psychological organization; (b) Prägnanz as a property of the result of such an organizational process; (c) Prägnanz steps as points of comparison when organizing the current stimulus; and (d) Prägnanz in relation to aesthetic appreciation (cf. Fig. 3).

More specifically, the law of Prägnanz concerns the tendency to achieve the best psychological organization possible given not only the visual input, but also the individual, the context, and their interactions. Importantly, the Prägnanz principle thus concerns experienced organizations — percepts — not stimuli. When the external stimulus factors are weak, the tendency towards Prägnanz can play a larger role. This tendency is realized by comparing the visual input to a (local or internal) reference, using two antagonistic but complementary tendencies. On the one hand unimportant differences are downsized or removed (i.e., leveling, simplification). On the other hand significant, characteristic differences are added or emphasized (i.e., sharpening, complication). Both tendencies contribute to the emergence of a better overall organization. Future research should investigate how these tension-reducing and tension-enhancing tendencies interact under different circumstances and clarify how the reference emerges.

For a psychological organization to be prägnant or ‘good’, the organization should be perceived as containing at least some form of unity or regularity, and, all other things being equal, Prägnanz increases when the organization is perceived as more autonomous, complete, simple of structure, element rich, expressive, and/or meaningful. Whereas some of these Prägnanz aspects relate to order and unity, other Prägnanz aspects relate to richness and intricacy (i.e., complexity). This highlights that both order and complexity play an important role in the concept of Prägnanz. Given these several aspects to Prägnanz, multiple Prägnanz tendencies can exist, and new research should illuminate which of these tendencies dominate under which conditions as well as how different tendencies interact.

When only one dimension of a stimulus is varied, the values on that dimension that are associated with the most prägnant percepts are called Prägnanz steps [*Prägnanzstufen*]. These Prägnanz steps will serve as reference levels for psychological organization: not only do they serve as a point of comparison for a broad range of percepts, they also make the organism more sensitive to change in their vicinity. The idea of prägnant Gestalts as reference points may however be useful in the multivariate case as well. When multiple stimulus dimensions are varied, the term Prägnanz height or dimensionality is used to indicate the Prägnanz or goodness of a phenomenally experienced organization. In the future, it is worthwhile to explore the diverse consequences of prägnant Gestalts and the interactions between those consequences in more detail and in a more systematic way than was done before, also taking the diverse Prägnanz aspects into account in the choice of the prägnant Gestalts under investigation.

Since the inception of Gestalt psychology, prägnant Gestalts have been proposed to be the percepts that are most aesthetically appreciated, in addition to the ones we tend to perceive when possible. That perception and appreciation are closely related is largely beyond doubt, but the exact relation between them is more difficult to pin down. Whether appreciation is related to the absolute Prägnanz level of a psychological organization, to the relative increase in Prägnanz and thus the strength of the experienced Prägnanz tendency, or to both is subject to further investigation. In addition, the result of these future investigations could depend on the Prägnanz aspects taken into account, so a systematic study of different possible combinations is recommended.

### General points of attention when investigating Prägnanz

One of the main goals of Gestalt psychology was to discover the principles governing human perceptual organization as well as the conditions influencing it (Ash, 1995; Koffka, 1935; Wertheimer, 1924/1999). Besides the above mentioned recommendations for future research related to the different uses of Prägnanz, some more general recommendations for future research on Prägnanz can be made.

#### Conduct concrete research with Prägnanz as a guiding principle

Let’s use Prägnanz and Gestalt theory more generally as Wertheimer (1924/1999) proposed it: as a framework and device for future, concrete research. Although most contemporary researchers have left the basic ideas of Prägnanz and Gestalt theory behind, we believe that these ideas can serve as important handles for improving our understanding of human psychological organization and visual perceptual organization in particular. As has been pointed out before (Ash , 1995; Smith , 1988; Wagemans, Feldman, et al., 2012), Gestalt theorists’ opposition to a simple associationist, elementaristic world view is a point that is still highly relevant in the current research climate. On the other hand, it is wrong to view Prägnanz as a fixed part of a theory to which no theoretical changes can be made, or as a finalized product that should be taken for granted or left aside. Further clarifications and specifications of the workings of the different Prägnanz tendencies in concrete cases are needed to further elaborate the general framework and test specific aspects of it.

#### Respect the richness and multiplicity of Prägnanz as a concept

Let’s not simplify Prägnanz to the narrow interpretations it has been given after having been taken out of its original Gestalt theoretical context. Especially, let’s consider that percepts and psychological organizations in general originate in organisms, which reveals that “goodness” of organization cannot be determined solely based on stimulus conditions, but needs to take into account the observer in its context. In different contexts, different Prägnanz aspects may become dominant (e.g., Marković & Gvozdenovi , 2001).

#### Investigate potential quantitative indicators of Prägnanz

Let’s use the diverse qualitative Prägnanz aspects defined by Rausch (1966) as a starting point for further reflection on different quantitative indicators of Prägnanz and quantitative models of Prägnanz tendencies. Quantifications have been proposed for some of the grouping principles and their interactions (e.g., proximity, similarity, good continuation; Claessens & Wagemans , 2008; Froyen et al., 2015; Jäkel et al., 2016; Kubovy & van den Berg , 2008; Kubovy & Wagemans , 1995; Quinlan & Wilton , 1998). Recently, individual differences in the strength of these grouping principles have also started to be taken into account (Van der Hulst et al. , 2023; Van Geert et al., 2022). Although each of these quantitative indicators in itself is not enough to replace the overall concept of Prägnanz, specifying the influences and interactions of different Prägnanz tendencies, quantitatively when possible, is the way forward proposed by the Gestalt psychologists themselves (Rausch, 1952; Wertheimer, 1924/1999).

#### Respect the qualitative version of Prägnanz

Let’s not overemphasize the importance of a definitive quantitative measure for Prägnanz. Although a quantitative indicator is helpful, not all useful concepts are easy to measure (and not all measures represent useful concepts).^16^ So, let’s not throw away the baby with the bathwater: Prägnanz is a valuable concept even when it cannot be quantitatively defined as an overarching concept. Koffka (1935) emphasized that although a quantitative formulation of Prägnanz is desirable, it only entails a more *precise* specification of the qualitative formulation, which is not different from it in kind. Rausch (1979/1992) also stressed the importance of paying attention to qualitative research methods and results next to quantitative ones.

#### Try to connect and compare Prägnanz to other perspectives on perception

Let’s try to see the connections between the Gestalt theoretical concept of Prägnanz and other perspectives on visual perception. Typically, ‘simplicity’ and ‘likelihood’ have been presented as contrasting principles (e.g., Pomerantz & Kubovy , 1986; van der Helm , 2000, cf. also Box 1), while overlap in ideas and predictions following from these ideas seems to have been largely ignored. Given that both Bayesian and Gestalt psychological views posit a connection to regularities in the physical world — be it directly by proposing veridicality or indirectly by proposing parallelism — it is understandable that both views will lead to similar results in many cases (cf. also van der Helm , 2000). Furthermore, Gestalt thought is not in contrast to any influence from learning or previous experience, rather to the contrary. The main difference is that Gestalt theory does not view ‘previous experience’ as a definitive answer to all questions of human perceptual organization, and it emphasizes the importance of more general principles of organization next to the influences of previous experience. A comparison, confrontation, or synthesis between these diverse views on perception can all advance research and unite the field. As Henle (1987) puts it, some researchers mainly see continuities (i.e., the dromedaries), others mainly see dichotomies (i.e., the camels). There are however many basic issues in psychology that cannot be solved by any of them. We need to overcome dichotomies in another way than by choosing one of the two, simply adding both, or finding a middle ground (Henle, 1987).

#### Look for potential neuroscientific indicators of structural energy

Let’s look at the neuroscientific processes underlying the principles of perceptual organization through the lens of Prägnanz. Can we find measures or indicators of structural energy across the brain? According to Köhler (1940), neuroscientific evidence is the only way to find support for the Prägnanz tendency as a tendency towards minimal structural energy. A study by Schurger et al. (2015) found a more stable neural activation pattern for trials in which a stimulus was consciously perceived compared to trials in which the stimulus was not consciously perceived. Although this study is not yet direct evidence for the Prägnanz tendency, it is congruent with Prägnanz as a tendency towards the best, most stable organization.

### Key takeaway

Prägnanz is a multifaceted Gestalt psychological concept indicating the “goodness” of a perceived organization. The stimulus constellation is not the only factor in determining the goodness of an organization, also the stimulus’ interaction with an individual in a specific spatial and temporal context plays a role.

The Prägnanz principle indicates a tendency present in every process of psychological organization to tend towards the most prägnant organization possible. As Prägnanz is a multifaceted concept, several tendencies can be present and their interaction in different stimuli, contexts, and individuals is an important area for further study.

Prägnanz as a concept cannot be reduced to a singular dimension, but entails diverse aspects including those related to order and unity as well as intricacy and complexity. Organizations particularly high in Prägnanz are sometimes used as a reference to which incoming stimuli are compared. In addition, Prägnanz has a close connection to aesthetic appreciation and artistic practice, although the exact relation is subject to further research.

Taking the ideas about Prägnanz as a guiding framework and keeping the original Gestalt psychological context in mind, future concrete research on perceptual organization can retake the path paved by the Gestalt psychologists by further specifying how different organizational principles interact in concrete situations, by respecting the nuanced and multifaceted nature of Prägnanz, and by clarifying which specific aspects of Prägnanz are under investigation.

Bringing different empirical findings together in a broader framework of tendency towards Prägnanz does not mean that it is not worth studying more specific effects, but integrating them in a broader framework will bring us from a lot of scattered pieces of information to knowledge of a system (see also Koffka , 1935).

## Data Availability

Data sharing is not applicable to this article as no datasets were generated or analysed during the current study. The review was not preregistered.

## Footnotes

1 In contrast to what many assume, Prägnanz is etymologically not related to the English word pregnancy (derived from the Latin word ‘praegnans’, meaning rich in potential content; Arnheim , 1975).

2 Koffka referred to this type of organization as ‘psycho-physical’ as the formation of these organizations is influenced by both internal (i.e., psychological) and external (i.e., physical) factors. Koffka’s notion should not be confused with Fechner’s notion of psychophysics, which concerns the quantitative mapping of physical stimuli onto psychological entities (e.g., between stimulus intensity and sensation strength).

3 Note that the terms we mention as approximately equivalent here have previously been used to indicate slightly different tendencies. For example, complication and simplification were defined as adding and removing parts, respectively (e.g., Fehrer , 1935; Hubbell , 1940). Sharpening, accentuation, and pointing were defined as emphasizing or exaggerating particularities of a figure, making the characteristics of a figure more differentiated (Fehrer, 1935; Hubbell, 1940; Wulf, 1922). The term leveling referred to downplaying particularities of a figure, making the characteristics of a figure less differentiated (Fehrer, 1935; Hubbell, 1940; Wulf, 1922). In addition, Wulf (1922) defined normalizing as increasing the resemblance to a familiar structure.

4 Although we do see the value of this distinction between Prägnanz tendencies, we do not think of these Prägnanz tendencies as *successive* processes in the way Kanizsa (1979) described primary and secondary processes. We believe that according to traditional Gestalt theory, there is not first an organization of the perceptual field and only then more high-level cognitive identification or classification, but rather one complex dynamic process of Gestalt formation (see also Kruse , 1986).

5 This also includes, for example, the effects of expertise (Koffka, 1935; Metzger, 1941, 1954, 1936/2006).

6 Although this distinction between properties may remind readers of Koffka ’s (1940) primary, secondary, and tertiary qualities, the classifications are not identical.

7 It must be clear that there is not a random relation between essence and structure: where the structure is, the essence is too (Metzger, 1941). The perfection of a poem, for example, lies in the fact that its meaning or essence becomes clear already in the sounds, not just in the words (Metzger, 1941). Another example could be the expressive fonts and typesetting used by Paul van Ostaijen, where the way of positioning the words and the fonts used already embody the meaning of the text. These mutual relationships between whole properties are not bidirectional, however: not for every possible structure [*Gefüge*] and also not for every possible material whole quality [*Beschaffenheit*] there is a special essence or being [*Wesen*] (Metzger, 1941).

8 As will become clear, this is in contrast with Rausch’s (1966) fifth Prägnanz aspect concerning element richness as contributing to a better overall organization or Gestalt.

9 This leaves the complexity or multiplicity aspects as indicated by Rausch (1966) — discussed under the section on “Rausch’s (1966) Prägnanz aspects” — untouched, however, and exclusively focuses on unity and simplicity as characteristic for Prägnanz.

10 Take note that the ‘structural complexity’ concept of Koenderink et al. (2018) does not refer to Rausch’s fourth Prägnanz aspect of the simplicity or complicatedness of structure, but rather to figural complexity in the sense of stimulus entropy or element richness, as in Rausch’s (1966) fifth Prägnanz aspect; cf. the section on “Rausch’s (1966) Prägnanz aspects” below.

11 An earlier English summary of Rausch’s (1966) ideas on the Prägnanz concept is available in Smith (1988).

12 This example is not given in Rausch (1966).

13 Just like derivations, disruptions can occur in form or shape, but also in position (i.e., location and orientation).

14 This example is not given in Rausch (1966).

15 Wertheimer (1923) presented the Prägnanz tendency under limited viewing conditions (i.e., assimilation to the prägnant form in tachistoscopic presentations) as the most extreme evidence for the statement that intermediate steps are psychologically perceived as “poorer” or “imperfect” versions of the Prägnanz steps.

16 As William Bruce Cameron (1963) said it: “Not everything that can be counted counts, and not everything that counts can be counted.”

## References

[CR1] Arnheim, R. (1975). Anwendungen gestalttheoretischer Prinzipien auf die Kunst [Applications of Gestalt theoretical principles to art]. In S. Ertel, L. Kemmler, & M. Stadler (Eds.), *Gestalttheorie in der modernen Psychologie [Gestalt theory in modern psychology]* (pp. 278–284). Darmstadt: Steinkopff. 10.1007/978-3-642-72312-4_28

[CR2] Arnheim R (1986). The two faces of Gestalt psychology. Am. Psychol..

[CR3] Arnheim, R. (1987). Prägnanz and its discontents. *Gestalt Theory*, *9*(2), 102–107.

[CR4] Ash, M. G. (1995). *Gestalt psychology in German culture, 1890–1967: Holism and the quest for objectivity*. Cambridge University Press. 10.1177/007327539803600404 11620555

[CR5] Attneave F (1954). Some informational aspects of visual perception. Psychol. Rev..

[CR6] Attneave F (1955). Symmetry, information, and memory for patterns. Am. J. Psychol..

[CR7] Biederman I (1987). Recognition-by-components: A theory of human image understanding. Psychol. Rev..

[CR8] Bischof N, Metzger W, Bergius R, Thomae H (1966). Erkenntnistheoretische Grundlagenprobleme der Wahrnemungspsychologie [Basic epistemological problems of the psychology of perception]. Allgemeine Psychologie [General psychology].

[CR9] Bock, H., & Pfeiffer, T. (1987). Prototypikalität von Bedeutungsvarianten des Verbs "überholen" im Lichte der gestalttheoretischen Bezugssystemlehre [Prototypicality of meaning variants of the Verb "to overtake" in the light of Gestalt theoretical frame of reference theory]. *Gestalt Theory,**9*(1), 3–16.

[CR10] Bosch, E., Fritsche, M., Ehinger, B. V., & de Lange, F. P. (2020). Opposite effects of choice history and evidence history resolve a paradox of sequential choice bias. *J. Vis.,**20*(12), 9. 10.1167/jov.20.12.9 10.1167/jov.20.12.9 PMC7683864 33211062

[CR11] Chater, N. (1996). Reconciling simplicity and likelihood principles in perceptual organization. *Psychol. Rev.,**103*(3), 566–581. 10.1037/0033-295X.103.3.566 8759047

[CR12] Checkosky SF, Whitlock D (1973). Effects of pattern goodness on recognition time in a memory search task. J. Exp. Psychol..

[CR13] Chetverikov, A., & Kristjánsson, Á. (2016). On the joys of perceiving: Affect as feedback for perceptual predictions. *Acta Psychol.,**169*, 1–10. 10.1016/j.actpsy.2016.05.005 10.1016/j.actpsy.2016.05.005 27195963

[CR14] Claessens PME, Wagemans J (2008). A Bayesian framework for cue integration in multistable grouping: Proximity, collinearity, and orientation priors in zigzag lattices. J. Vis..

[CR15] Clement DE (1964). Uncertainty and latency of verbal naming responses as correlates of pattern goodness. J. Verbal. Learn. Verbal. Behav..

[CR16] Clement DE, Varnadoe KW (1967). Pattern uncertainty and the discrimination of visual patterns. Percept. Psychophys..

[CR17] Ellis WD (1938). A source book of Gestalt psychology.

[CR18] Eysenck, H. J. (1942). The experimental study of the ‘good Gestalt’–a new approach. *Psychol. Rev.,**49*(4), 344–364. 10.1037/h0057013

[CR19] Fehrer EV (1935). An investigation of the learning of visually perceived forms. Am. J. Psychol..

[CR20] Feldman J (2000). Bias toward regular form in mental shape spaces. J. Exp. Psychol. Hum. Percept. Perform..

[CR21] Feldman J (2003). What is a visual object?. Trends Cogn. Sci..

[CR22] Froyen V, Feldman J, Singh M (2015). Bayesian hierarchical grouping: Perceptual grouping as mixture estimation. Psychol. Rev..

[CR23] Garner WR (1974). The processing of information and structure.

[CR24] Glanzer M, Clark WH (1963). Accuracy of perceptual recall: An analysis of organization. J. Verbal Learn. Verbal Behav..

[CR25] Goldmeier E (1937). Über Ähnlichkeit bei gesehenen Figuren [About similarity in seen figures]. Psychol. Forsch..

[CR26] Goldmeier, E. (1972). Similarity in visually perceived forms. *Psychol. Issues,**8*(1), 1–136. 4661205

[CR27] Goldmeier E (1982). The memory trace: Its formation and its fate.

[CR28] Graf, L. K. M., & Landwehr, J. R. (2015). A dual-process perspective on fluency-based aesthetics: The Pleasure-Interest Model of Aesthetic Liking. *Personal. Soc. Psychol. Rev.,**19*(4), 395–410. 10.1177/1088868315574978 10.1177/1088868315574978 25742990

[CR29] Graf LKM, Landwehr JR (2017). Aesthetic pleasure versus aesthetic interest: the two routes to aesthetic liking. Front. Psychol..

[CR30] Hendrickx, M., & Wagemans, J. (1999). A critique of Leyton’s theory of perception and cognition. Review of *Symmetry, Causality, Mind,* by Michael Leyton. *J. Math. Psychol.**43*(2), 314–345. 10.1006/jmps.1998.1232

[CR31] Henle M (1987). On breaking out of dichotomies. Gestalt Theory.

[CR32] Hochberg J (1968). Perception.

[CR33] Hochberg J (2003). Acts of perceptual inquiry: Problems for any stimulus-based simplicity theory. Acta Psychol..

[CR34] Hochberg, J., & McAlister, E. (1953). A quantitative approach, to figural "goodness". *J. Exp. Psychol.,**46*(5), 361–364. 10.1037/h0055809 10.1037/h0055809 13109140

[CR35] Hoffman, D. D. (2009). The interface theory of perception: Natural selection drives true perception to swift extinction. In S. J. Dickinson, A. Leonardis, B. Schiele, & M. J. Tarr (Eds.), *Object categorization: Computer and human vision perspectives* (pp. 148–166). Cambridge: Cambridge University Press. 10.1017/CBO9780511635465.009

[CR36] Hoffman DD, Singh M, Prakash C (2015). The interface theory of perception. Psychon. Bull. Rev..

[CR37] Hubbell MB (1940). Configurational properties considered ‘good’ by naïve subjects. Am. J. Psychol..

[CR38] Hüppe A (1984). Prägnanz - ein gestalttheoretischer Grundbegriff: Experimentelle Untersuchungen [Prägnanz - a basic concept in gestalt theory: Experimental investigations].

[CR39] Jäkel F, Singh M, Wichmann FA, Herzog MH (2016). An overview of quantitative approaches in Gestalt perception. Vis. Res..

[CR40] Kanizsa, G. (1975). "Pragnanz" as an obstacle to problem-solving. *G. Ital. Psicol.,**2*, 417–425.

[CR41] Kanizsa G (1979). Organization in vision: Essays on Gestalt perception.

[CR42] Kanizsa G, Luccio R (1986). Die Doppeldeutigkeiten der Prägnanz [The ambiguities of Prägnanz]. Gestalt Theory.

[CR43] Koenderink J (2014). The All Seeing Eye?. Perception.

[CR44] Koenderink J (2015). Esse est percipi & verum factum est. Psychon. Bull. Rev..

[CR45] Koenderink J (2019). Vision, an Optical User Interface. Perception.

[CR46] Koenderink J, van Doorn A, Pinna B (2018). Measures of Prägnanz?. Gestalt Theory.

[CR47] Koffka K (1935). Principles of Gestalt psychology.

[CR48] Koffka K, Bernheimer R (1940). Problems in the psychology of art. Art: A Bryn Mawr symposium.

[CR49] Köhler W (1920). Die physischen Gestalten in Ruhe und im stationären Zustand [The physical Gestalten at rest and in stationary state].

[CR50] Köhler, W. (1940). *Dynamics in psychology*. Liveright.

[CR51] Köhler, W. (1993). Letter to Abraham S. Luchins (December 6, 1951). "... The principle of Prägnanz is probably in need of a revised formulation...". *Gestalt Theory*, *15*(3–4), 297–298. (Original work published 1951).

[CR52] Kruse, P. (1986). Wie unabhängig ist das Wahrnehmungsobjekt vom Prozeß der Identifikation: Ein Kommentar zu G. Kanizsa und R. Luccio [How independent is the perceptual object from the process of identification: A comment on G. Kanizsa and R. Luccio]. *Gestalt Theory**8*(2), 141–143.

[CR53] Kubilius J, Sleurs C, Wagemans J (2017). Sensitivity to nonaccidental configurations of two-line stimuli. I-Perception.

[CR54] Kubilius J, Wagemans J, Op de Beeck HP (2014). Encoding of configural regularity in the human visual system. J. Vis..

[CR55] Kubovy M, van den Berg M (2008). The whole is equal to the sum of its parts: A probabilistic model of grouping by proximity and similarity in regular patterns. Psychol. Rev..

[CR56] Kubovy M, Wagemans J (1995). Grouping by proximity and multistability in dot lattices: A quantitative Gestalt theory. Psychol. Sci..

[CR57] Leeuwenberg ELJ, Boselie F (1988). Against the likelihood principle in visual form perception. Psychol. Rev..

[CR58] Leeuwenberg ELJ, van der Helm PA (1991). Unity and variety in visual form. Perception.

[CR59] Leeuwenberg ELJ, van der Helm PA (2012). Structural Information Theory: The simplicity of visual form. Cambridge University Press.

[CR60] Leyton M (1992). Symmetry, causality, mind.

[CR61] Luccio R (2019). Perceptual simplicity: The true role of Prägnanz and Occam. Gestalt Theory.

[CR62] Luchins AS, Luchins EH (1998). Commentary on Vicario’s “On Wertheimer’s principles of organization”. Gestalt Theory.

[CR63] Marković S, Gvozdenovi V (2001). Symmetry, complexity and perceptual economy: Effects of minimum and maximum simplicity conditions. Vis. Cogn..

[CR64] Metzger, W. (1941). *Psychologie: Die Entwicklung ihrer Grundannahmen seit der Einführung des Experiments [Psychology: The development of its basic assumptions since the introduction of the experiment]*. Berlin, Heidelberg: Springer-Verlag. Retrieved from 10.1007/978-3-642-53395-2

[CR65] Metzger, W. (1954). Grundbegriffe der Gestaltpsychologie [Fundamental concepts of Gestalt psychology]. In J. de Ajuriaguerra, et al. (Ed.), *Aktuelle Probleme der Gestalttheorie [Current problems in Gestalt theory]*. Bern: Huber.

[CR66] Metzger W, Metzger W, Bergius R, Thomae H (1966). Figural-wahrnemung [Figural perception]. Allgemeine Psychologie [General psychology].

[CR67] Metzger, W. (1975). *Gesetze des Sehens**[Laws of seeing]* (3rd ed.). Frankfurt a. M.: Kramer.

[CR68] Metzger, W. (2006). *Laws of seeing*. Cambridge, MA: MIT Press. (Original work published 1936).

[CR69] Muth C, Carbon C-C (2013). The Aesthetic Aha: On the pleasure of having insights into Gestalt. Acta Psychol..

[CR70] Muth, C., & Carbon, C.-C. (2016). SeIns: Semantic instability in art. *Art Percept.,**4*(1–2), 145–184. 10.1163/22134913-00002049

[CR71] Muth C, Pepperell R, Carbon C-C (2013). Give me Gestalt! Preference for cubist artworks revealing high detectability of objects. Leonardo.

[CR72] Palmer SE, Beck J (1982). Symmetry, transformation, and the structure of perceptual systems. Organization and representation in perception.

[CR73] Palmer, S. E. (1991). Goodness, Gestalt, groups, and Garner: Local symmetry subgroups as a theory of figural goodness. In G. R. Lockhead & J. R. Pomerantz (Eds.), *The perception of structure: Essays in honor of Wendell R. Garner* (pp. 23–39). Washington, DC: American Psychological Association. 10.1037/10101-001

[CR74] Pascucci D, Mancuso G, Santandrea E, Libera CD, Plomp G, Chelazzi L (2019). Laws of concatenated perception: Vision goes for novelty, decisions for perseverance. PLoS Biol..

[CR75] Pascucci D, Tanrikulu ÖD, Ozkirli A, Houborg C, Ceylan G, Zerr P, Kristjänsson Á (2023). Serial dependence in visual perception: A review. J. Vis..

[CR76] Pepperell, R. (2018). Art, energy, and the brain. In J. F. Christensen & A. Gomila (Eds.), *Progress in Brain Research* (Vol. 237, pp. 417–435). Elsevier. 10.1016/bs.pbr.2018.03.022 10.1016/bs.pbr.2018.03.022 29779747

[CR77] Petermann B (1931). Das Gestaltproblem in der Psychologie im Lichte analytischer Besinnung: Ein Versuch zu grundsätzlicher Orientierung [The Gestalt problem in psychology in the light of analytical reflection: An attempt at fundamental orientation].

[CR78] Peterson MA, Gibson BS (1994). Object recognition contributions to figure-ground organization: Operations on outlines and subjective contours. Percept. Psychophys..

[CR79] Pomerantz JR (1977). Pattern goodness and speed of encoding. Mem. Cogn..

[CR80] Pomerantz JR, Garner WR (1973). The role of configuration and target discriminability in a visual search task. Mem. Cogn..

[CR81] Pomerantz, J. R., & Kubovy, M. (1986). Theoretical approaches to perceptual organization: Simplicity and likelihood principles. In *Handbook of perception and human performance, Vol. 2: Cognitive processes and performance*. (pp. 1–46). Oxford, England: John Wiley & Sons.

[CR82] Post, R. A. G., Blijlevens, J., & Hekkert, P. (2016). "To preserve unity while almost allowing for chaos": Testing the aesthetic principle of unity-in-variety in product design. *Acta Psychol.,**163*, 142–152. 10.1016/j.actpsy.2015.11.013 10.1016/j.actpsy.2015.11.013 26687018

[CR83] Prasad D, Bainbridge WA (2022). The visual Mandela effect as evidence for shared and specific false memories across people. Psychol. Sci..

[CR84] Quinlan PT, Wilton RN (1998). Grouping by proximity or similarity? Competition between the Gestalt principles in vision. Perception.

[CR85] Rausch, E. (1952). *Struktur und Metrik figural-optischer Wahrnehmung [Structure and metrics of figural-optical perception]*. Frankfurt a. M.: Verlag Dr. Waldemar Kramer.

[CR86] Rausch E, Metzger W, Bergius R, Thomae H (1966). Das Eigenschaftsproblem in der Gestalttheorie der Wahrnemung [The property problem in the Gestalt theory of perception]. Allgemeine Psychologie [General psychology].

[CR87] Rausch, E. (1979/1992). Neun Wünsche an die Zukunft der Psychologie (Auszugsweiser Nachdruck eines 1979 erschienenen Gesprächs mit E. Rausch) [Nine wishes for the future of psychology (Excerpt reprint of a conversation with E. Rausch published in 1979)]. *Gestalt Theory*, *14*(2), 143–144.

[CR88] Rogers B (2014). Delusions about Illusions. Perception.

[CR89] Rosch, E. (1973). On the internal structure of perceptual and semantic categories. In T. E. Moore (Ed.), *Cognitive development and acquisition of language* (pp. 111–144). San Diego: Academic Press. 10.1016/B978-0-12-505850-6.50010-4

[CR90] Rosch E (1975). Cognitive reference points. Cogn. Psychol..

[CR91] Rosch E, Rosch E, Lloyd BB (1978). Principles of categorization. Cognition and categorization.

[CR92] Sablé-Meyer M, Fagot J, Caparos S, van Kerkoerle T, Amalric M, Dehaene S (2021). Sensitivity to geometric shape regularity in humans and baboons: A putative signature of human singularity. Proc. Natl. Acad. Sci..

[CR93] Sadil, P., Cowell, R., & Huber, D. E. (2021). *The push-pull of serial dependence effects: Attraction to the prior response and repulsion from the prior stimulus*. PsyArXiv. 10.31234/osf.io/f52yz 10.3758/s13423-023-02320-3 PMC11488665 37566217

[CR94] Schumann, F. (1914). *Bericht über den VI. Kongreß für experimentelle Psychologie in Göttingen vom 15. Bis 18. April 1914 [Report on the VI Congress of Experimental Psychology in Güttingen from April 15 to 18, 1914]*. Leipzig: Verlag von Johann Ambrosius Barth.

[CR95] Schurger A, Sarigiannidis I, Naccache L, Sitt JD, Dehaene S (2015). Cortical activity is more stable when sensory stimuli are consciously perceived. Proc. Natl. Acad. Sci..

[CR96] Sheehan, T. C., & Serences, J. T. (2023). *Distinguishing response from stimulus driven history biases*. bioRxiv. 10.1101/2023.01.11.523637

[CR97] Smith B (1988). Foundations of Gestalt theory.

[CR98] Sorge, S. (1940). Neue Versuche über die Wiedergabe abstrakter optischer Gebilde [New experiments on the reproduction of abstract optical formations]. *Arch. Gesamte Psychol.,**106*, 1–88.

[CR99] Spröte P, Schmidt F, Fleming RW (2016). Visual perception of shape altered by inferred causal history. Sci. Rep..

[CR100] Stadler M, Stegnano L, Trombini G (1979). Quantitative Analyse der Rauschschen Prägnanzaspekte [Quantitative analysis of Rausch’s Prägnanz aspects]. Gestalt Theory.

[CR101] Strother, L., & Kubovy, M. (2006). On the surprising salience of curvature in grouping by proximity. *J. Exp. Psychol. Hum. Percept. Perform.,**32*(2), 226–234. 10.1037/0096-1523.32.2.226 10.1037/0096-1523.32.2.226 16634667

[CR102] Strother, L., & Kubovy, M. (2012). Structural salience and the nonaccidentality of a gestalt. *J. Exp. Psychol. Hum. Percept. Perform.,**38*(4), 827–832. 10.1037/a0027939 10.1037/a0027939 22486306

[CR103] Sundqvist, F. (2003). *Perceptual dynamics: Theoretical foundations and philosophical implications of Gestalt psychology* (PhD thesis). Göteborg University; Acta Universitatis Gothoburgensis, Göteborg, Sweden.

[CR104] Van de Cruys S, Wagemans J (2011). Putting reward in art: A tentative prediction error account of visual art. I-Perception.

[CR105] van der Helm PA (2000). Simplicity versus likelihood in visual perception: From surprisals to precisals. Psychol. Bull..

[CR106] van der Helm PA (2017). On Bayesian simplicity in human visual perceptual organization. Perception.

[CR107] Van der Hulst, E., van Heusden, E., Wagemans, J., & Moors, P. (2023). Additivity of grouping by proximity and luminance similarity is dependent on relative grouping strength: An analysis of individual differences in grouping sensitivity. *Atten. Percept. Psychophys.*10.3758/s13414-023-02770-w 10.3758/s13414-023-02770-w 37740153

[CR108] Van Geert, E., Bossens, C., & Wagemans, J. (2023). The Order & Complexity Toolbox for Aesthetics (OCTA): A systematic approach to study the relations between order, complexity, and aesthetic appreciation. *Behav. Res. Methods,**55*, 2423–2446. 10.3758/s13428-022-01900-w 10.3758/s13428-022-01900-w 36171524

[CR109] Van Geert E, Frérart L, Wagemans J (2023). Towards the most prägnant Gestalt: Leveling and sharpening as contextually dependent adaptive strategies. Mem. Cogn..

[CR110] Van Geert E, Moors P, Haaf J, Wagemans J (2022). Same stimulus, same temporal context, different percept? Individual differences in hysteresis and adaptation when perceiving multistable dot lattices. I-Perception.

[CR111] Van Geert, E., & Wagemans, J. (2020). Order, complexity, and aesthetic appreciation. *Psychol. Aesthet. Creat. Arts,**14*(2), 135–154. 10.1037/aca0000224

[CR112] Van Geert E, Wagemans J (2023). What good is goodness? The effects of reference points on discrimination and categorization of shapes. J. Exp. Psychol. Hum. Percept. Perform..

[CR113] van Lier, R., van der Helm, P. A., & Leeuwenberg, E. L. J. (1994). Integrating global and local aspects of visual occlusion. *Perception,**23*(8), 883–903. 10.1068/p230883 10.1068/p230883 7870565

[CR114] von Ehrenfels C (1916). Höhe und Reinheit der Gestalt [Height and purity of Gestalt]. Kosmogonie [Cosmogony].

[CR115] von Ehrenfels C (1922). Das Primzahlengesetz, entwickelt und dargestellt auf Grund der Gestalttheorie [The prime number law, developed and presented on the basis of the Gestalt theory].

[CR116] von Ehrenfels, C. (1937). *Über Gestaltqualitäten (1932)* [On Gestalt qualities (1932)]. *Philosophia (Belgrad)*, *2*, 139–141. (Original work published 1932).

[CR117] Wagemans J (1992). Perceptual use of nonaccidental properties. Can. J. Psychol..

[CR118] Wagemans, J. (2015). Historical and conceptual background: Gestalt theory. In J. Wagemans (Ed.), *The Oxford handbook of perceptual organization*. Oxford University Press. 10.1093/oxfordhb/9780199686858.013.026

[CR119] Wagemans, J. (2018). Perceptual organization. In *Stevens’ handbook of experimental psychology and cognitive neuroscience: Vol. 2. sensation, perception, and attention*. John Wiley & Sons, Inc. 10.1002/9781119170174.epcn218

[CR120] Wagemans, J., Elder, J. H., Kubovy, M., Palmer, S. E., Peterson, M. A., Singh, M., & von der Heydt, R. (2012). A century of Gestalt psychology in visual perception: I. Perceptual grouping and figure–ground organization. *Psychol. Bull.**138*(6), 1172–1217. 10.1037/a0029333 10.1037/a0029333 PMC3482144 22845751

[CR121] Wagemans, J., Feldman, J., Gepshtein, S., Kimchi, R., Pomerantz, J. R., van der Helm, P. A., & van Leeuwen, C. (2012). A century of Gestalt psychology in visual perception: II. Conceptual and theoretical foundations. Psychol. Bull., *138*(6), 1218–1252. 10.1037/a0029334 10.1037/a0029334 PMC3728284 22845750

[CR122] Wagemans J, Vanden Bossche P, Segers N, d’Ydewalle G (1994). An affine group model and the perception of orthographically projected planar random polygons. J. Math. Psychol..

[CR123] Wellek, A. (1959). Das Prägnanzproblem der Gestaltpsychologie und das "Exemplarische" in der Pädagogik [The problem of Prägnanz in Gestalt psychology and the "exemplary" in pedagogy]. *Z. Exp. Angew. Psychol.,**6*, 722–736.

[CR124] Wertheimer M (1912). Über das Denken der Naturvölker. I. Zahlen und Zahlgebilde [About the thinking of people who live close to nature. I. Numbers and number formations.]. Z. Psychol..

[CR125] Wertheimer M (1922). Untersuchungen zur Lehre von der Gestalt. I. Prinzipielle Bemerkungen [Investigations into the teachings of Gestalt. I. Remarks on its principles]. Psychol. Forsch..

[CR126] Wertheimer M (1923). Untersuchungen zur Lehre von der Gestalt. II [Investigations into the teachings of Gestalt. II]. Psychol. Forsch..

[CR127] Wertheimer M (1959). Productive thinking.

[CR128] Wertheimer, M. (1999). Gestalt theory. *Gestalt Theory*, *21*, 181–183. (Original work published 1924).

[CR129] Wertheimer, M., Spillmann, L., Sarris, V., & Sekuler, R. (2012). *On perceived motion and figural organization*. Cambridge, MA: MIT Press.

[CR130] Wulf, F. (1922). Beiträge zur Psychologie der Gestalt. VI. Über die Veränderung yon Vorstellungen (Gedächtnis und Gestalt) [Contributions to the Psychology of Gestalt. VI. On the change of ideas (Memory and Gestalt)]. *Psychol. Forsch.*, *1*(1), 333–373. 10.1007/BF00410394

[CR131] Zimmer, A. C. (1991). The complementarity of singularity and stability. A comment on Kanizsa & Luccio’s "Analysis of the concept of Prägnanz" (1986). *Gestalt Theory*, *13*(4), 276–282.

